# Exploring evidence gaps in clinical trials in thermal burns care: an umbrella review

**DOI:** 10.1136/bmjopen-2024-094303

**Published:** 2025-06-25

**Authors:** Hollie Sarah Richards, Riaz Qureshi, Suzannah Kinsella, Sarah Dawson, Robert Staruch, Alice Lee, Jill Meirte, Janine Evans, Krissie Stiles, Niall Martin, Baljit Dheansa, Jane Blazeby, Jelena Savović, Amber Young

**Affiliations:** 1 NIHR Bristol Biomedical Research Centre, University of Bristol Medical School, Bristol, UK; 2 Department of Ophthalmology, University of Colorado School of Medicine, Aurora, Colorado, USA; 3 James Lind Alliance, Southampton, UK; 4 Population Health Sciences, Bristol Medical School, University of Bristol, Bristol, UK; 5 Oxford University Hospitals NHS Foundation Trust, Oxford, UK; 6 Academic Department of Military Surgery and Trauma, Royal Centre for Defence Medicine, Birmingham, UK; 7 St Andrew’s Centre for Plastic Surgery and Burns, Mid and South Essex NHS Foundation Trust, Basildon, UK; 8 Department of Rehabilitation Sciences and Physiotherapy, University of Antwerp, Antwerpen, Belgium; 9 Organisation for Burns Scar After-Care and Research, Antwerp, Belgium; 10 The Welsh Centre for Burns & Plastic Surgery, Abertawe Bro Morgannwg University Health Board, Swansea, UK; 11 Plastic Surgery, King’s College Hospital NHS Foundation Trust, London, UK; 12 Chelsea and Westminster Hospital NHS Foundation Trust, London, UK; 13 Department of Surgery and Cancer, Imperial College London, London, UK; 14 Department of Plastic Surgery and Burns, Queen Victoria Hospital NHS Foundation Trust, East Grinstead, UK; 15 NIHR Applied Research Collaboration West (ARC West) at University Hospitals Bristol and Weston NHS Foundation Trust, Bristol, UK

**Keywords:** Systematic Review, INTENSIVE & CRITICAL CARE, SURGERY

## Abstract

**Abstract:**

**Background:**

Gaps in research evidence lead to research waste. In burns treatment, there is a paucity of reliable evidence or data. This contributes to inconsistent patient care, especially on a global scale, where low-resource countries often lack access to the latest research advancements. This umbrella review was undertaken as part of the James Lind Alliance Priorities in Global Burns Research Prioritisation Setting Partnership (PSP) and aimed to identify and assess the quality of evidence in thermal burns care. The objective was to map which interventions in thermal burns care are supported by a reliable evidence base and for which the evidence is lacking.

**Methods:**

Systematic reviews of randomised controlled trials in thermal burns were identified and assessed using reliability criteria determined a priori. Multiple systematic review databases were searched in June 2023, including the Cochrane Library, KSR Evidence database and NIHR Journals Library. Summary of findings and, where available, Grading of Recommendations Assessment, Development and Evaluation was used to assess certainty of evidence. Reliable reviews were mapped onto clinical categories identified by patients, carers and healthcare professionals as part of the PSP.

**Results:**

232 systematic reviews were identified, of which 83 met reliability criteria and were included. The main reason for not meeting reliability criteria was poorly defined eligibility criteria (n=128). Of the 83 reliable reviews, most were conducted in pain (n=28) or wound management (n=14) and acute care (n=13). Certainty of evidence was mixed. Reviews mapped onto nine of the 17 clinical categories identified by the PSP.

**Conclusion:**

This review summarises the available high-quality evidence in burns care and identifies evidence gaps, indicating that many important clinical questions remain unanswered. There is a discrepancy between the treatments investigated in high-quality research and the clinical areas considered as most important to stakeholders. These findings provide direction for future research to improve global burns care.

**STRENGTHS AND LIMITATIONS OF THIS STUDY**

- This is a comprehensive umbrella review of reliable systematic reviews in burns care that provides a description of evidence for many interventions.
- This evidence has been compared against research priority questions codeveloped with burn survivors, carers and healthcare professionals as part of a James Lind Alliance Prioritisation Setting Partnership, illustrating evidence gaps in the literature.
- However, only English language papers were included in this review, meaning that findings may not be fully representative of global burns research.
- As only systematic reviews of randomised controlled trials (RCTs) were included, more discrete burns interventions that may not be suitable for RCTs or included in systematic reviews may be excluded from the findings.

## Introduction

 Systematic and transparent methods to identify and prioritise gaps in healthcare research are needed to inform funding strategies.^1 2^ Research gaps exist when it is not possible to draw a conclusion for a particular question or treatment because of insufficient evidence.^3^ These gaps often persist due to resource constraints, poor quality studies or heterogeneity in outcome reporting^4^ and because studies may not address relevant research questions that are important to patients and clinicians.^5^ Different methods can be used to identify health research gaps and priorities, including systematic and scoping reviews, evidence gap mapping, quantitative surveys and qualitative investigations.^6^

In the context of burns treatment, research gaps contribute to the wide disparity in care in burn services, within countries and internationally.^7^,^9^ Some evidence mapping has been conducted in wound debridement,^10^ paediatric burns^11^ and health systems,^9^ but there is a paucity of high-quality research in international burns care.^11 12^ These evidence gaps are especially relevant in the context of low-resource settings (eg, low- and middle-income countries (LMIC)) where 70% of global burn injuries occur.^13^,^15^ In addition to the higher incidence of burns injury in these areas, access to specialist burns care is limited by economic and geographical constraints,^15^ meaning that treatment is often delayed or inadequate.^11^ In the absence of an adequate evidence base, there is a lack of consensus on effective burns treatments. In terms of health systems research in burns care, only 13% of studies identified in a recent review were conducted in low-resource settings.^9^

To address these issues, an international James Lind Alliance (JLA) research prioritisation project was undertaken.^16^ In accordance with standardised JLA methodology,^17^ the Priorities in Global Burns Research Prioritisation Setting Partnership (PSP) used consensus methods with key stakeholders to identify the top 10 research priorities. Initial work explored the experiences of burns survivors, carers and healthcare professionals (HCPs) with online surveys and semistructured interviews. A long list of summary questions was developed relating to the aspects of acute and long-term thermal burns care most important to stakeholders. To determine which of these questions represented genuine gaps in the literature, it was necessary to examine the published evidence which typically focuses on randomised controlled trials (RCTs) and evidence synthesis of trials. Given the breadth of research areas represented by the Global Priorities in Burns Research PSP, this was not practical. As a high level of evidence was needed to determine whether the summary questions were already unanswered, an umbrella review (which identifies all systematic reviews of RCTs) was undertaken.

The aim of this review was to identify, summarise and appraise the quality of evidence of interventions in thermal burns care to establish which interventions are supported by a reliable evidence base and for which the evidence is lacking.

## Methods

### Eligibility criteria

We included reliable systematic reviews of RCTs of burn interventions. Specific eligibility criteria based on population, intervention, comparator and outcomes (PICO) are described in more detail in box 1. We defined ‘systematic reviews’ as (1) full-text reports that self-reported their methods as systematic reviews or meta-analyses anywhere in the text or (2) those which met the Institute of Medicine definition (‘a scientific investigation that focuses on a specific question and uses explicit, pre-specified scientific methods to identify, select, assess and summarise the findings of similar but separate studies’).^18^ We included systematic reviews of RCTs evaluating the effectiveness of interventions for thermal burns. Systematic reviews that included both RCTs and non-randomised studies of intervention were included only if separate synthesis of RCTs was available and extractable. We excluded diagnostic, prognostic, aetiological/risk factors and burden of disease reviews. For eligible systematic reviews with more than one publication (eg, updated Cochrane reviews or co-publications of Cochrane reviews in other journals), we included the most recent or the most complete version of the report. As part of the selection process, we assessed the reliability of reviews (see *Reliability assessment*).

Box 1 **Specific eligibility criteria of systematic reviews**

**Specific eligibility criteria**

- *Participants:* thermal burns patients and survivors of all ages, genders and nationalities. Electrical, chemical and radiation burns were excluded (see protocol^16^).
- *Interventions*: any interventions for treating patients who have experienced thermal burns. This included interventions for direct treatment of burns and their consequences and any other types of burns care, but also psychological interventions designed to help patients live with the long-term consequences of burns.
- *Comparators*: any comparator was included.
- *Outcomes*: all outcomes relating to prehospital, hospital or outpatient burns care were included.
- *Study designs*: systematic reviews of randomised trials of interventions in burns care.

### Bibliographic database searches and study selection

We developed a search strategy using terms that identify thermal burns interventions and systematic reviews to search the following systematic review databases in June 2023: Cochrane Library; KSR Evidence database (https://ksrevidence.com); NIHR Journals Library; International Network of Agencies for Health Technology Assessment; Epistemonikos; Health Evidence DoPHER. Additionally, we searched for systematic reviews in Ovid Medline and Ovid Embase as general medical bibliographic databases. The full search strategies are provided in online supplemental file 1.

We worked with the information specialist (SD) to conduct the searches to identify all systematic reviews published within the previous 5 years. Titles and abstracts were screened for relevancy by one researcher (HRS) using a sensitive and inclusive approach within the Rayyan database (https://www.rayyan.ai/). Relevant full-text articles were independently assessed for eligibility by two members of the team (HRS and RQ) using the PICO Portal web platform (www.picoportal.org). We resolved discrepancies through discussion.

### Data extraction

We conducted data extraction in two stages. Initially, we assessed included reviews for reliability according to criteria determined a priori (see Reliability assessment). The reviews meeting these assessment criteria progressed to full data extraction. We designed and piloted a custom reliability assessment form on PICO Portal. Data extraction was conducted on the Systematic Review Data Repository Plus (https://srdrplus.ahrq.gov/) web platform.

#### Reliability assessment

The reliability assessment and electronic data extraction form was adapted from one developed by the Cochrane Eyes and Vision United States Project and used by several guideline committees—including the European Glaucoma Society and the American Academy of Ophthalmology—to assess reviews of interventions.^19 20^ Reliability was assessed by one researcher (RQ) who is experienced in assessment and was one of the lead developers of the reliability assessment criteria. The reliability assessment was developed as a brief checklist to critically appraise the methods and conclusions presented in a systematic review. Reliability items were derived from the Critical Appraisal Skills Programme,^21^ the Assessment of Multiple Systematic Reviews^22^ and the Preferred Reporting Items for Systematic Reviews and Meta-Analyses.^23^ A copy of the data extraction form is available in online supplemental appendix 1. Following the assessment, a review is considered reliable if it meets the following five criteria:

1. Defined eligibility criteria for selection of individual studies.
2. Conducted a comprehensive literature search for eligible studies.
3. Assessed the risk of bias of the individual included studies using any method.
4. Used appropriate methods for meta-analyses (criterion will only be assessed if meta-analysis was performed).
5. We observe concordance between the review findings and conclusions.

Each of these five criteria is considered critical to a review being ‘reliable’ and useful for informing clinical practice. Thus, a systematic review of interventions is considered ‘unreliable’ when one or more of these criteria were not met. If a review did not meet these criteria, no further data extraction was undertaken. Specific definitions of the five criteria are adapted from Qureshi *et al*^20^ and are described in table 1.

**Table 1:** Criteria for assessing the reliability of systematic reviews

Criterion	Definition applied to systematic review reports
Defined eligibility criteria	Described inclusion and/or exclusion criteria for eligible studies.
Conducted comprehensive literature search	Review authors: (1) described an electronic search of two or more bibliographic databases; (2) used a search strategy comprising a mixture of controlled vocabulary and keywords; (3) reported using at least one other method of searching such as searching of conference abstracts; (4) identified ongoing trials; (5) complemented electronic searching by hand search methods (eg, checking reference lists); (6) contacted the included study authors or experts.
Assessed risk of bias of included studies	Used any method (eg, scales, checklists or domain-based evaluation) designed to assess methodologic rigour of included studies.
Used appropriate methods for meta-analysis	Used quantitative methods that (1) were appropriate for the study design analysed (eg, maintained the randomised nature of trials; used adjusted estimates from observational studies); (2) correctly computed the weight for included studies.
Observed concordance between review findings and conclusions	Authors’ reported conclusions were consistent with findings, provided a balanced consideration of benefits and harms and did not favour a specific intervention if there was lack of evidence.

Our classification of the reliability of systemic reviews of interventions was based on information from the methods reported in the publication and involved judgement; we did not review protocols or contact review authors to obtain additional information. We did not evaluate or assess the methodology used by the reviewers (eg, the method used to assess risk of bias in individual studies). Furthermore, our classification of reliability focused solely on five criteria applied to the systematic review report and not the quality or certainty of the body of evidence. For example, a reliable systematic review may find low-quality evidence or no evidence. As such, the reliability of reviews defined by our five criteria and covered in the report should not be confused with an assessment of the certainty of evidence for burns treatment.

#### Full data extraction

For the reviews that met the reliability criteria, we extracted the objective and main conclusion of the systematic review, review characteristics including PICO and the clinical area of the intervention (eg, surgery, rehabilitation, wound management). If provided in the review, we extracted a summary of findings and Grading of Recommendations Assessment, Development and Evaluation (GRADE) assessments/ratings of the body of evidence, but we did not undertake assessments of body of evidence ourselves. Data extraction was conducted by one researcher (HSR) and verified by a second researcher (RQ).

#### Mapping the identified evidence and evidence gaps against the priorities identified by the Priorities in Global Burns Research PSP

Reviews were further classified by intervention type and categorised in accordance with the categories determined from qualitative analysis of data from the first survey of the Priorities in Global Burns Research (PSP).^16^ These 17 categories included improving wound management, improving rehabilitation and reducing pain and reflected the issues reported as being most important to burns patients, their carers and HCPs through online surveys and interviews (online supplemental appendix 2). A review could be associated with more than one category (eg, a review assessing reducing pain specifically in the context of wound management). Within each category, reviews were mapped against the 52 ‘unanswered question’ priorities identified by the PSP. For example, all reviews with a focus on pain reduction in burns care were allocated to the reducing pain category and mapped against the ‘unanswered question’ priority, ‘what are the best ways to understand and manage pain and anxiety from burn injuries and treatments to improve care?’ An ‘unanswered question’ would be considered to be fully answered if a systematic review made clear conclusions about the effectiveness of an intervention that was the primary topic of an ‘unanswered question’. Members of the research team (HSR and JB) and six burns clinicians assessed each mapped review to determine whether the ‘unanswered question’ had been fully answered by the review.

### Patient and public involvement

Patients and the public were not directly involved in this umbrella review. However, as part of the wider project, five members of the steering group with patient and public involvement and engagement representatives were directly involved in the analysis that produced the priorities to which this review’s findings were mapped to in order to identify evidence gaps.

## Results

### Searches and study selection

**Figure 1:**
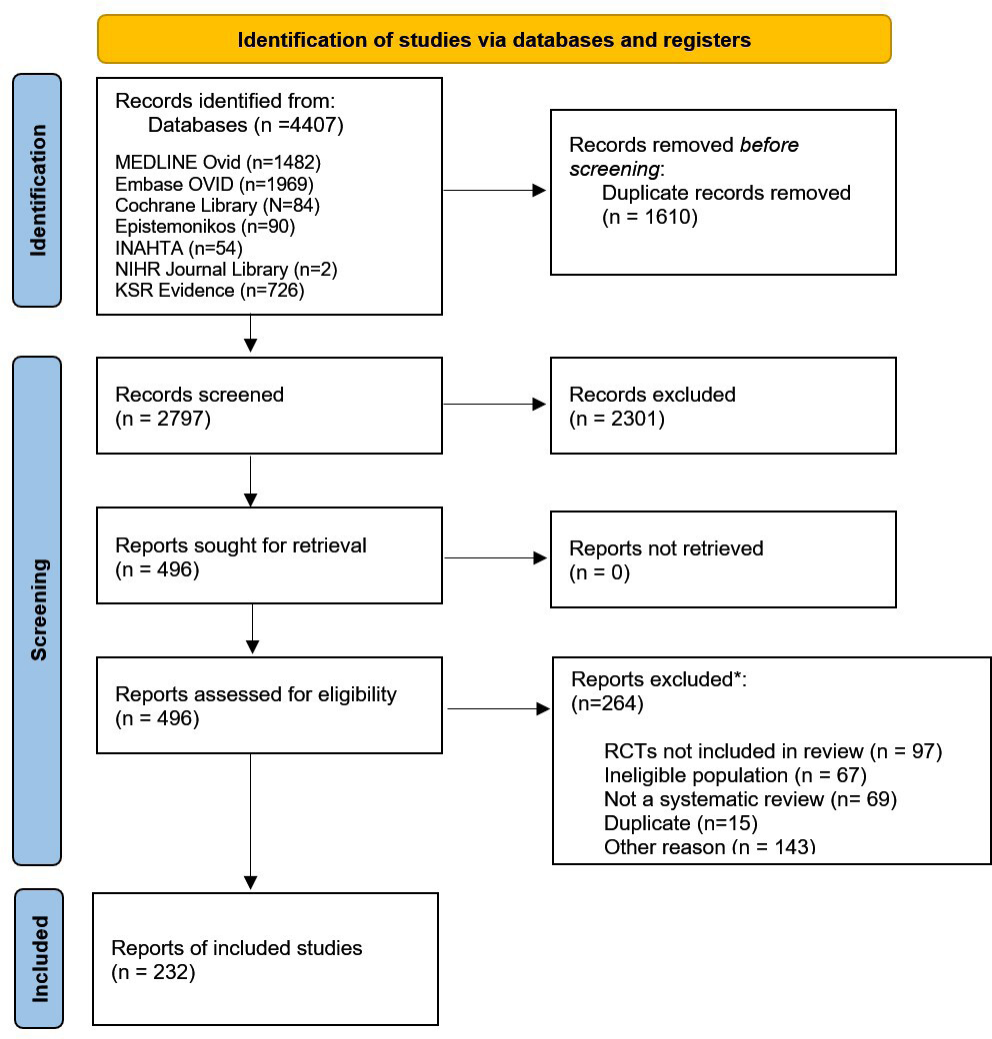
Preferred Reporting Items for Systematic Reviews and Meta-Analyses diagram. *Records could be excluded for multiple reasons at full-text level. INAHTA, International Network of Agencies for Health Technology Assessment; RCT, randomised controlled trial.

Searches of 7 databases identified 4407 reviews, 2797 of which were retained after deduplication. Following screening of titles/abstracts and full texts, a total of 232 reviews were included and assessed for reliability. An overview of searches and study selection is displayed in figure 1.

### Characteristics of reviews

Of the 232 reviews that were assessed for reliability, 83 (36%) met the criteria and progressed to full data extraction. Of these, the average number of RCTs relating to burns treatments in included reviews was 8 (range 1–37). The majority of reviews evaluated pain management (n=28, 34%), wound management (n=14, 17%) and resuscitation and acute management (n=13, 16%). 15 (18%) reviews focused on adult patients, 16 (19%) on paediatric populations, 17 (21%) were mixed populations and 35 (42%) were unclear (eg, ‘burns patients’). 22 (27%) reviews included studies from LMIC status countries; 25 (30%) only included studies from high-income countries, and for 36 (43%) reviews, it was unclear whether LMIC status countries were included. Online supplemental table 1 includes detailed study characteristics.

#### Reliability assessment

**Figure 2:**
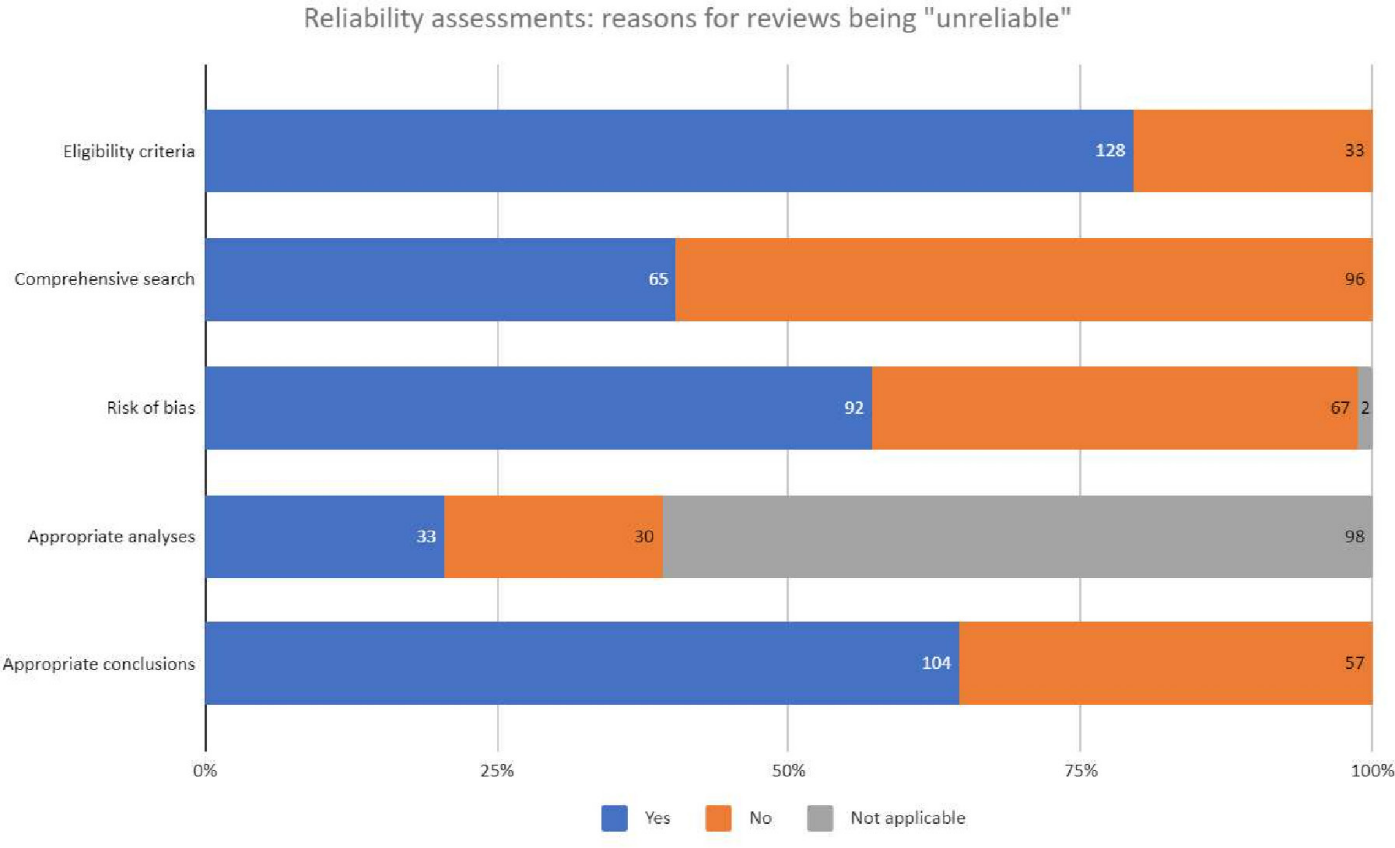
Distribution of reasons for reviews being found ‘unreliable’ following reliability assessments.

The main reasons for the 149 excluded reviews not meeting the reliability assessment criteria were poorly defined eligibility criteria (n=128, 55%), lack of concordance between conclusion and findings (n=104, 45%) and poor risk of bias assessments (n=92, 40%). 65 (28%) reviews were considered ‘unreliable’ due to the lack of comprehensive searches, and 33 (14%) did not conduct appropriate analysis. 61 (26%) reviews were considered ‘unreliable’ based on one criterion, 48 (21%) did not meet two criteria and 27 (12%) did not meet three criteria. Figure 2 shows the distribution of reasons for reviews being found ‘unreliable’, and an overview of the reliability assessment is provided in online supplemental file 4.

### Overview of evidence

The 83 reviews covered a wide range of interventions and outcomes. We classified reviews for synthesis according to their purpose (ie, reducing pain, wound management, early resuscitation and management, rehabilitation, reducing scarring, improving surgical techniques, improving psychosocial outcomes and preventing burns). Many types of interventions were used across multiple of these themes, showing their breadth of application. The most commonly studied types of interventions included pharmaceuticals, virtual reality and behavioural adjustment techniques, and approaches to debridement and wound cleaning/dressing.

#### Interventions to reduce pain

28 reviews evaluated approaches to reduce pain. Of these, 10 reviews were focused on children and/or adolescents and six reviews on adults, with the remaining 12 either including all ages or not specifying age. Virtual reality and digital distraction techniques were the most commonly assessed, with 12 reviews examining these types of interventions specifically and nine (75%) of these finding significant effects on reducing pain.^24^,^32^ Five reviews studied pharmacological interventions to reduce pain, and three (60%) of these found significant and strong evidence of an effect.^33^,^35^ Five reviews examined the effectiveness of other non-digital distraction techniques such as aromatherapy, massage or hypnosis as the primary interventions of interest, and all found significant improvements in reducing management of pain and anxiety, although the evidence from these reviews was more mixed and limited by few primary studies.^36^,^40^ Six reviews studied a broad range of non-pharmacological interventions, and four (66%) of these found significant and positive benefits to reducing pain.^41^,^44^

#### Interventions to improve wound management

14 reviews assessed interventions to improve wound management, of which one was in children, one in adults and 12 did not specify any age restrictions. These reviews studied a variety of interventions including pharmacological interventions, different types of dressings and surgical techniques (eg, approaches to debridement) for outcomes like pain and quality of life, as well as complications, graft characteristics and wound appearance (ie, scar texture, colour and healing). The evidence for these interventions was more mixed and inconclusive, compared with the reviews of interventions to reduce pain. Of three reviews comparing debridement techniques, two found ‘promising’ evidence of a significant effect for enzymatic debridement on reducing the need for autografting and wound outcomes.^10 45^ Of the four reviews of pharmacological agents (eg, platelet rich plasma, bromelain, collagenase, papain, sutilain, antibiotics and oxandolone) applied topically or injected to the wound site, two (50%) found significant positive effects on wound healing for platelet rich plasma and oxandolone^46 47^ and one (25%) found a negative effect, concluding that systemic antibiotic prophylaxis was ‘inefficient’ in preventing infections in paediatric burns.^48^ Similarly, for different types of wound dressings, four of seven (57%) reviews found positive effects^49^,^52^ and one (14%) presented evidence that wounds dressed with silver sulfadiazine had similar healing as without, but that burns treated without silver were at lower risk of reinfection than ones treated with silver.^53^

#### Interventions for early resuscitation and management

13 reviews assessed interventions for early resuscitation and management, four were in adults, one in children, three in adults and children and five did not specify age. The most commonly assessed type of intervention was nutritional support (eg, different enteral nutrition regimens and glutamine supplementation) with four reviews, three of which found significant improvements in patient outcomes such as mortality, complications, organ failure and length of hospital stay with glutamine supplementation and starting enteral support early.^54^,^56^ Fluid replacement strategies were studied in three reviews with mixed evidence, as one found no difference in effect between colloids and crystalloids on mortality,^57^ one found a promising but inconclusive effect of hyperosmotic fluids^58^ and one found colloids (hypo-oncotic albumin solution) led to more increased pulmonary fluid than hyperoncotic albumin solution.^59^ Of two reviews studying beta-blockers, one found limited evidence to make conclusions^60^ and one found a reduction in mortality among critically ill patients.^61^ One review was found for each of ventilation strategies,^62^ cool running water over burns,^63^ transfusion volumes^64^ and programmes to address palliative care needs,^65^ of which only 20 min of cool running water within 3 hours of the injury had sufficient evidence for the authors to make a clear recommendation for its use.

#### Interventions to improve rehabilitation

Eight reviews examined approaches to improving rehabilitation (four in adults, one in children and three without age restrictions), seven of which focused on different types of exercises, the use of orthoses or movement therapies and one on diet. Nearly all of the reviews on exercises found significant and positive effects on aspects such as physical function/mobility, quality of life, burn healing and pain, although all reported generally low levels of evidence that increase the uncertainty of these findings.^66^,^71^ The one review of high protein diet for improving the nutritional status and subsequent clinical outcomes of burns patients found only weak evidence to support administering high protein diets to patients following burns.^72^

#### Interventions to reduce scarring

Seven reviews studied interventions to reduce scarring. None of the reviews had any age restrictions. Three reviews studied forms of electrical stimulation (eg, transcutaneous electrical nerve stimulation or extracorporeal shock wave therapy), two of which found significant positive effects.^73 74^ One review examined whether fluid silicone gels positively affect scar quality and incidence and found clear significant improvements.^75^ One review studied different types of ablative lasers, but found too much heterogeneity to reach any conclusions about effectiveness.^76^ Two reviews studied a variety of interventions including pressure garments, gel sheeting, exercises, pressure therapy, massage, CO_2_ laser and lotion with massage, but both concluded that most interventions needed more evidence to support recommendations.^77 78^

#### Interventions to improve surgical techniques

Six reviews assessed different surgical techniques and add-ons to improve the effects of surgical interventions, of which one was in adults and five did not have age restrictions. Two reviews assessed skin substitutes (eg, Biobrane, TransCyte, Integra, Glyaderm, Suprathel, Apligraft, Duoderm, Bacitracin, Allograft and autologous skin cell suspension) and found significant positive benefits in treating wounds.^79 80^ One assessed a similar type of intervention for corneal burns and found uncertain evidence of any positive effect on epithelialisation or visual outcomes.^81^ Two reviews examined topical agents during surgery to control intraoperative bleeding and found the evidence for all types (eg, fibrin glue, phenylephrine, epinephrine and tranexamic acid) to be inconclusive due to limited number of studies and poor quality.^82 83^ One study of early versus late excision or grafting concluded early intervention is associated with improvements in mortality and patient-important outcomes, but that their conclusions were made with low certainty.^84^

#### Interventions to improve psychosocial outcomes

Four reviews studied interventions to improve psychosocial outcomes in burn patients; two were focused on children and adolescents, and two had no age restrictions. These reviews studied either exclusively different therapy techniques (eg, cognitive behavioural therapy, art therapy, counselling, ‘healing touch’, etc) or a mix of psychosocial therapies and pharmacologics (eg, propanol, sertraline, haloperidol, zolpidem, etc). The evidence was mixed, with three of the reviews finding evidence that the non-pharmacological therapies were effective in reducing pain and anxiety and improving sleep quality.^85^,^87^ However, two reviews found the evidence for sleep-promotion interventions to be sparse and inconsistent, with low to very-low certainty.^86 88^ One of the reviews that included pharmacologics found that medications were not effective in reducing stress symptoms.^87^

#### Interventions to prevent burns

Three reviews studied approaches to burn prevention, of which one focused on children in LMIC and two did not have age restrictions. These reviews all assessed changes in knowledge and found telehealth interventions using smartphone apps were useful for widespread and targeted awareness campaigns, but acknowledged that changes in understanding do not necessarily equate to changes in rates of incidents.^89^,^91^ Two reviews on education initiatives concluded there was not enough evidence to determine whether education can impact burn incidence.^90 91^

### GRADE, summary of findings and ‘certainty’ of evidence

Of the 83 reliable reviews, 18 reported using GRADE to assess the certainty of the evidence. One review did not provide a summary of findings table and may have incorrectly used GRADE to judge the quality of the included studies, noting ‘the overall quality of all included studies was low according to the GRADEPro software’.^43^ The other 17 reviews included at least one summary of findings table, overall, reporting the certainty of evidence for a total of 207 outcomes. The certainty of evidence was ‘very low’ for 101 (49%) outcomes, ‘low’ for 41 (20%), ‘moderate’ for 14 (7%) and ‘high’ for 6 (3%). The certainty was not reported for 45 (22%) outcomes, which is typically indicated when there was no evidence found to support an outcome. Most outcomes were downgraded for more than one reason; the most common reasons were risk of bias (n=127) and imprecision (n=106). All extracted GRADE assessments from the 17 reviews are available in online supplemental file 2.

The 20 outcomes with moderate or high certainty evidence came from four reviews. The six outcomes with ‘high’ certainty all came from Heliste *et al*,^61^ which examined mortality and cardiovascular outcomes among critically ill patients treated with beta-blockers. Heliste *et al*^61^ also found two of its outcomes to have ‘moderate’ certainty. Of the 12 other ‘moderate’ certainty outcomes, 10 came from Lewis *et al*,^57^ which assessed mortality and renal outcomes across four different comparisons of colloids and crystalloids for fluid resuscitation among critically ill patients. Hoggewerf *et al*^92^ found ‘moderate’ certainty evidence for the use of topical antibiotics for facial burns for the outcome time-to-wound-healing. McQuilten *et al*^64^ found ‘moderate’ certainty evidence for the comparison of 1:1:1 vs 1:1:2 of fresh frozen plasma:platelets:red blood cells transfusion ratios on the rate of complications.

### Mapping the identified evidence and evidence gap with the priorities identified by the Priorities in Global Burns Research PSP

Of the 83 reliable reviews, 82 mapped onto at least one ‘unanswered question’ identified by the Priorities in Global Burns Research PSP, whereby the interventions described by the review directly related to the topic of the ‘unanswered question’. The review that did not relate to an ‘unanswered question’ focused specifically on amniotic membrane transplantation for ocular burns, which is a topic that did not feature in the generation of ‘unanswered questions’ from our survey data. Assessments to determine if any of the systematic reviews fully answered the ‘unanswered questions’ were undertaken by one researcher (HSR), three burns clinicians (JM, JE and KS) and three burns surgeons (RS, NM and AL). This was done by examining the PICO, objectives and conclusions of each review in relation to the mapped ‘unanswered questions’. For example, one systematic review investigating the prophylactic use of systemic antibiotics in preventing infectious complications in paediatric burns^48^ was assessed against the ‘unanswered question’, ‘what are the best ways of preventing, identifying, diagnosing and treating burn wound infections and sepsis?’ In each instance, it was determined that no reviews fully answered any of the ‘unanswered questions’ generated from survey data (see online supplemental file 3, Evidence checking).

The 17 categories comprising the 52 ‘unanswered questions’ represent the experiences, issues and questions most important to burns patients, survivors, their carers and HCPs. Systematic reviews focused on interventions relating to nine (53%) of these categories, most commonly around reducing pain (n=28, 34%), improving wound management (n=23, 28%), improving burns resuscitation and early management (n=14, 17%), improving rehabilitation (n=9, 11%) and improving scarring (n=7, 8%). The number of reviews that mapped to burns care categories is presented in table 2. Of the 52 ‘unanswered questions’, systematic reviews assessed interventions that related to 25 (48%) questions. Further information is provided in online supplemental file 3, Evidence checking.

**Table 2:** Mapping of reviews to clinical questions

Categories of burns care most important to stakeholders* (n=17)	Number of reviews of interventions relating to category (n=82)†
Reducing pain	28 (34%)
Improving wound management	23 (28%)
Improving burns resuscitation and early management	14 (17%)
Improving rehabilitation	9 (11%)
Improving scarring	7 (8%)
Improving surgical interventions for burns	7 (8%)
Improving psychosocial outcomes	4 (5%)
Burns prevention	3 (4%)
Clinician and patient interactions and communications	1 (1%)
Managing long-term issues and chronic morbidities related to burn injuries	0
Optimising access to treatment	0
Improving clinician and patient education around burns treatment	0
Inhalation injury	0
Optimising the timings of burns treatments	0
Resources	0
Clinician’s well-being	0
Developing new treatments and standardising care	0

* Categories were generated from analysis of responses to the Priorities in Global Burns Research Prioritisation Setting Partnership first survey (n=1617) and interviews (n=16).^16^

† Systematic reviews may address more than one category.

## Discussion

### Summary of findings

This umbrella review has identified systematic reviews in burns treatments. Of 232 records, 83 (36%) were included. Of these, the three clinical areas with most research were pain (n=28, 34%), wound management (n=23, 28%) and resuscitation/acute care (n=14, 17%). Some evidence was found for the effectiveness of non-pharmacological pain treatments, such as virtual reality and distraction techniques. There was evidence to support the use of exercise-based rehabilitation techniques, some scar reduction treatments (eg, electrical stimulation and silicone gel) and skin substitutes. The evidence for wound management, resuscitation and acute care was of mixed strength, as were reviews that examined psychological interventions for anxiety related to pain, sleep quality and stress. This umbrella review therefore allowed the evidence gaps in burns treatment to be delineated. Based on the priorities identified by stakeholders in earlier work,^93^ the main areas of need for future research are psychosocial interventions, management of long-term recovery and improving education of burns treatments among clinicians.

The 17 areas of research into which the umbrella review findings were mapped have been identified and prioritised by patients and clinicians as part of the wider PSP project.^93^ Nearly all reviews (82/83) matched against nine of the clinical categories; one review was focused on ocular burns which we deemed too distinct from the general wound management category. Interestingly, eight of these categories did not have reliable reviews of RCT evidence associated with them. Although some of these categories may be difficult to address by RCT approaches (eg, optimising access to treatment, resources or developing new treatments), other categories such as long-term morbidities or inhalation injuries would be very suitable.

High-quality evidence-based clinical practice is key in achieving parity in burns care internationally and across diverse healthcare economies. As a specialty, burns is in a transitionary phase as it aims to develop more high-quality evidence to support clinical practice.^94^,^96^ To achieve this, an appraisal of what high-level evidence and systematic reviews are currently available is a necessary first step. It is also a crucial part of the research prioritisation process as it contextualises the priorities against the current research gaps within the field.^17^ This has important implications for research, both in bottom-up study design and top-down funding prioritisation. This review can prompt that process by neatly presenting the key research gaps within the field in a systematic fashion for the first time. It also presents a critical reminder of the limitations of currently available evidence. This has immediate short-term clinical implications, primarily updating clinicians on the availability of high-quality evidence in key domains of their clinical practice, thereby ensuring the delivery of evidence-based care. The longer-term implications of this are, as of yet, unquantifiable, as this work lays the groundwork for more focused research programmes.

### Implications for research

We are not aware of any other overviews of burns interventions with the scope and purpose that we set out to achieve. The current reliable evidence identified in this review addresses only nine of the 17 categories identified as most important to survivors, carers and HCPs and reveals contradictory findings within the evidence. Additionally, only four of the reviews reported moderate or high certainty of evidence. Although progress has been made in early management of burns patients with a reduction in mortality,^97^ the evidence from this umbrella review indicates that many questions remain unanswered. These include specific fluid resuscitation regimens, debridement techniques (eg, surgical vs non-surgical and timing of excision), selection of skin substitutes, use of topical haemostatic agents for intraoperative bleeding and effective wound dressings. For example, skin substitute products are expanding in number and uptake, without high-quality evidence to guide product selection, resulting in non-standardised care. In this review, no records were mapped to inhalational injuries, which is a significant mortality predictor.^98^ This finding highlights an evidence gap which needs addressing urgently. Given that only 83 of 232 reviews met reliability criteria and there was general low quality of evidence, future researchers should consider methodological recommendations highlighted by limitations in the included systematic reviews; for example, designing better powered studies, in different global settings and higher quality reporting. This will reduce research waste, prevent patients from being involved in poor quality research that will not benefit others and can ensure robust, reproducible results.

### Implications for practice

Although the evidence was generally of low certainty and there are many clinical questions that remain unanswered, there was sufficient evidence to make recommendations on some topics, for example, the use of cool running water for 20 min shortly after injury. Further worldwide implementation of these first aid principles seems necessary. Reviews of pain management interventions suggest that virtual reality and digital distraction techniques may be effective, although this technology is less accessible in low-resource settings. Overall, fewer reviews were mapped to topics relating to chronic burns sequelae such as rehabilitation and scar management. In burn rehabilitation, active exercise treatments increasingly focus on early intervention, starting at day 1. These include interventions for improving physical activity with exercise and resistance training or splints and casts for retaining mobility and function. Reviews focussing on rehabilitation found positive results but with low certainty of evidence. From a clinical perspective, it is surprising that pressure therapy/garments and scar massage were not more widely addressed by reliable reviews. Treatments for the prevention and reduction of pathological scar formation identified in this review tended to focus on more advanced technology (eg, electrical stimulation and laser treatments), which are increasingly used but lack high-quality evidence of effectiveness.^99^

From prehospital admission to long-term follow-up, burns care includes a variety of interventions undertaken by multiple teams of HCPs. Treatment objectives change over time depending on individual patient/carers needs and availability of resources and staff. Feasibility and implementation of some interventions in this review remain unclear, with some higher cost interventions (eg, laser therapy, skin substitutes and virtual reality devices for pain distraction) likely to be more difficult to implement in lower resource settings, regardless of evidence level. Further research, including qualitative approaches, may be needed to further explore these implications. In terms of acceptability, there may be scepticism towards interventions which deviate from the standard medical model, for example, using non-pharmacological modalities for pain management (aromatherapy, massage and hypnosis) or non-surgical debridement techniques. Treatments such as physical therapy for rehabilitation and scar management, massage, shockwave and other mechanical treatments are already established practice globally based on anecdotal evidence and are therefore unlikely to change despite low-level evidence in the literature. Such treatments are challenging to adequately evaluate for effectiveness, in part because they are not linked to industry and require complex large-scale research projects, meaning funding is harder to secure. As these non-pharmacological interventions are generally low cost and low risk, it may be beneficial to continue their use in some settings (eg, LMIC) until the evidence becomes clearer.

Development of new treatments in the burns field is welcome but should be supported by methodologically strong evidence to standardise care; indeed, in this review, no evidence was mapped to standardisation of treatment. Future research needs to be methodologically robust, address known research uncertainties and ultimately lead to more standardised care.

### Strengths and limitations

Previous work has examined evidence gaps in health systems research^9^ and paediatric burns care.^11^ To our knowledge, this is the first comprehensive review of high-quality systematic reviews in burns care that provides a description of evidence for many interventions in burns and compares this against the priority questions agreed through stakeholder consensus, illustrating areas where evidence is missing (evidence gaps). However, there are a number of limitations. Only English language papers were included in this overview, meaning that findings may not be representative of global burns research, although it is considered unlikely that important findings were missed as only 13% of global research is conducted in lLMICs,^9^ and well-designed and conducted studies are likely to have been translated and cited. Only systematic reviews were included, meaning that more discrete burns interventions that are potentially not included in any systematic reviews were excluded from the findings. We did not check for primary studies’ being included multiple times across overlapping systematic reviews, which could lead to some evidence being ‘double counted’.^100^ However, the range of interventions and settings in which they were applied makes it unlikely that potential overlap would have affected our conclusions. Similarly, only evidence from RCTs was included in the analysis, and we did not update reviews with more recent RCTs. We did not search for primary studies or individual RCTs in clinical areas not represented in identified systematic reviews. It is worth noting that some relevant areas of research may not be methodologically suitable for RCTs and may therefore be under-represented. Additionally, we did not assess the certainty of evidence if it was not provided in reviews, and we only summarised it where it was available. Our searches may not have been sensitive enough to comprehensively identify evidence gaps in the global burns literature. To identify potential gaps in evidence, initial broad searches should ideally be supplemented with targeted searches focused on specific interventions or clinical areas, identified a priori*,* usually with guidance from stakeholders. Although our searches covered all aspects of burns care, we did not undertake additional targeted searches for the clinical categories identified from the wider work of the Priorities in Global Burns Research PSP. These searches had been planned in the original protocol,^16^ but this was not possible due to reduced team capacity following the death of the principal investigator.

## Conclusion

This umbrella review summarises high quality systematic review evidence in burn care. Most reviews focus on pain management, wound care, resuscitation and acute care. When the findings were mapped against the broad treatment priorities identified by stakeholders, evidence for nine of the 17 burns treatments was found. However, some had contradictory findings. Of particular interest, this review highlights that many key areas of clinical care lack high-quality evidence. These include specific fluid resuscitation regimens, debridement techniques, skin substitutes, use of topical haemostats, wound dressing and care of the inhalation injury. These findings, together with the top 10 research priorities identified by the PSP,^93^ provide direction for future research to ensure it is addressing treatments of most importance to patients and clinicians.

## Supplementary material

10.1136/bmjopen-2024-094303 online supplemental file 1

10.1136/bmjopen-2024-094303 online supplemental file 2

10.1136/bmjopen-2024-094303 online supplemental file 3

10.1136/bmjopen-2024-094303 online supplemental file 4

10.1136/bmjopen-2024-094303 online supplemental table 1

10.1136/bmjopen-2024-094303 online supplemental appendix 1

10.1136/bmjopen-2024-094303 online supplemental appendix 2

## Data Availability

All data relevant to the study are included in the article or uploaded as supplementary information.
