# Supplementary material for: Exploring evidence gaps in clinical trials in thermal burns care: an umbrella review
Source: BMJ Open. 2025 Jun 25;15(6):e094303. doi: 10.1136/bmjopen-2024-094303 (PMC12198850; doi:10.1136/bmjopen-2024-094303)
Supplement: online supplemental file 1 [file bmjopen-15-6-s001.docx]

**Burns Review**

Summary of numbers:

- **MEDLINE Ovid** (2012 to 25 January 2023), n=1453 [Batch-1]
- **MEDLINE Ovid** (25 January 2023 to 15-March-2023), n=29
- **Embase Ovid** (2018 to 15 March 2023), n=1969 [Batch-2 (29 (MED) + 1094 (Embase) new)]
- **Cochrane Library** (CENTRAL & CDSR) (all years to 19 Apr 2023), n=84 [Batch-3 (47 new)]
- **Epistemonikos** (all available years to 27 Apr 2023), n=90 [Batch-4 (24 new)]
- **Health Evidence** (all available years 27 Apr 2023) (0)
- **DoPHER** (all available years to 27 Apr 2023) (0)
- **INAHTA** (all available years to 31-May-2023) (54) [Batch-5 (52 new)]
- **NIHR Journals Library** (all years to 31-May-2023) (2) [Batch-6 (2 new)]
- **KSR Evidence** (all available years to 8 June 2023) (726) [Batch-7 (96 new)]

Total=4407

Duplicates=1610

Uploaded to Rayyan=2797

************************************************************************

Ovid MEDLINE(R) ALL <1946 to January 25, 2023>

[Date limited 2012 onwards]

1 burns/ or eye burns/ 50861

2 (burn or burns or burned or postburn* or scald*).ti,ab,kf. 74542

3 (burn or burns).jw. 15819

4 (thermal adj (injur* or wound*)).ti,ab,kf. 5888

5 burns, inhalation/ or smoke inhalation injury/ 2419

6 ((smoke adj3 inhal*) and (injur* or trauma* or wound?)).ti,ab,kf. 1002

7 or/1-6 89184

8 "systematic review"/ or meta-analysis/ or network meta-analysis/ 299694

9 (systematic or structured or evidence or trials or studies).ti. and ((review or overview or look or examination or update* or summary).ti. or review.pt.) 312035

10 (0266-4623 or 1469-493X or 1366-5278 or 1530-440X or 2046-4053).is. 20783

11 meta-analysis.pt. or (meta-analys* or meta analys* or metaanalys* or meta synth* or meta-synth* or metasynth*).ti,ab,kf,hw. 292109

12 ((systematic adj2 analys*) or (meta adj2 (analys* or review))).ti,kf. 182922

13 (((systematic* or quantitativ* or methodologic*) adj5 (review* or overview*)) or (quantitativ* adj5 synthes*)).ti,ab,kf,hw. 335849

14 (quantitativ* adj3 (analys* or evaluat*)).ti,ab,kf. and ((review or overview or look or examination or update* or summary).ti. or review.pt.) 11154

15 (integrative research review* or research integration).tw. or scoping review?.ti,kf. or (review.ti,kf,pt. and (trials as topic or studies as topic).hw.) or (evidence adj3 review*).ti,ab,kf. 235502

16 review.pt. and ((medline or medlars or embase or pubmed or scisearch or psychinfo or psycinfo or psychlit or psyclit or cinahl or electronic database* or bibliographic database* or computeri#ed database* or online database* or pooling or pooled or mantel haenszel or peto or dersimonian or der simonian or fixed effect or ((hand adj2 search*) or (manual* adj2 search*))).tw,hw. or (retraction of publication or retracted publication).pt.) 206285

17 or/8-16 759789

18 7 and 17 1994

19 exp animals/ not humans.sh. 5086667

20 18 not 19 1952

21 limit 20 to yr="2012 -Current" 1453

[Updated 1-March &^-^ 15-March 2023 (+29)]

Key to search fields

ti:title; ab:abstract; kf:author keyword; jw:journal word; pt:publication type; hw:subject heading word

************************************************************************

Ovid Embase <1974 to 2023 March 15>

[Date limited 2018 onwards]

1 burn.hw. 82958

2 (burn or burns or burned or postburn* or scald*).ti,ab,kf. 88654

3 (burn or burns).jw. 21969

4 (thermal adj (injur* or wound*)).ti,ab,kf. 7724

5 injury/ and inhalation/ 1036

6 ((smoke adj3 inhal*) and (injur* or trauma* or wound?)).ti,ab,kf. 1400

7 or/1-6 119554

8 systematic review/ or meta analysis/ or network meta-analysis/ 551810

9 ((systematic or structured or evidence or trials or studies) and (review or overview or look or examination or update* or summary)).ti. 304178

10 (0266-4623 or 1469-493X or 1366-5278 or 1530-440X or 2046-4053).is. 25076

11 (systematic review? or evidence report* or technology assessment?).jw. 38221

12 (meta-analys* or meta analys* or metaanalys* or meta synth* or meta-synth* or metasynth*).ti,ab,kw,hw. 440498

13 ((systematic or meta) adj2 (analys* or review)).ti,kw. or ((systematic* or quantitativ* or methodologic*) adj5 (review* or overview*)).ti,ab,kw,sh. or (quantitativ* adj5 synthes*).ti,ab,kw,hw. 578939

14 exp "clinical trial (topic)"/ and review.ti,kw,pt. 176489

15 (integrative research review* or research integration).ti,ab,kw. or scoping review?.ti,kw. or (evidence adj3 review*).ti,ab,kw. 92750

16 review.pt. and (medline or medlars or embase or pubmed or scisearch or psychinfo or psycinfo or psychlit or psyclit or cinahl or electronic database* or bibliographic database* or computeri#ed database* or online database* or pooling or pooled or mantel haenszel or peto or dersimonian or der simonian or fixed effect or ((hand adj2 search*) or (manual* adj2 search*))).ti,ab,kw,hw. 226636

17 review.pt. and ((evidence based adj (medicine or practice)) or (outcome? adj (assessment or research)) or treatment outcome).hw. 253165

18 or/8-17 1130113

19 7 and 18 4401

20 limit 19 to yr="2018 -Current" 1969

********************************************************************************

**Cochrane Library**

Issue 4 of 12, 2023

Searched: 19 April 2023

#1 (burn or burns or burned or postburn* or scald*):ti,ab,kw 6166

#2 (thermal NEXT (injur* or wound*)):ti,ab,kw 404

#3 ((smoke NEAR inhal*) and (injur* or trauma* or wound*)):ti,ab,kw 55

#4 #1 OR #2 OR #3 6427

Limited to Database of Systematic Reviews (CDSR): Reviews (77); Protocols (7)

************************************************************************

**Epistemonikos**

Searched: 27 Apr 2023

Systematic Reviews > Interventions:

#1 (burn OR burns OR burned OR postburn* OR scald*) (86)

#2 (thermal* AND (injur* or wound*)) (10)

#3 (smoke AND inhal*) (3 (not relevant)

#4 (#1 OR #2) (90)

************************************************************************

**NIHR Journals Library** (31-May-2023)

<https://www.journalslibrary.nihr.ac.uk/search/#/>

S1: *burn* > Limited to Evidence Synthesis (20)

Relevant records saved (2)

S2: *burns* (15)

Relevant records saved (2 (duplicates))

S3: *burned* (0)

S4: *postburn* or *postburns* or *postburned* (0)

S5: "*thermal injury*" OR "*thermal injuries*" (2)

Relevant records saved (0)

S6: "*thermal wound*" OR "*thermal wounds*" (0)

S7: *smoke inhalation (*39)

Relevant records saved (0)

S8: *smoke AND inhal* (*0)

S9: HRCS Health Category: Injuries and Accidents (119)

Records screened in-situ for relevant reviews (0)

OR/S1-S9 (2)

************************************************************************

**International Network of Agencies for Health Technology Assessment** (INAHTA) [https://database.inahta.org](https://database.inahta.org/)

Searched: 31-May-2023

S1 "Burns"[mh](23)

S2 ((burn or burns or burned or postburn* or scald*))[Title] OR ((burn or burns or burned or postburn* or scald*))[abs] (51)

S3 ("thermal injury" OR "thermal injuries" OR "thermal wound" OR "thermal wounds")[Title] OR ("thermal injury" OR "thermal injuries" OR "thermal wound" OR "thermal wounds")[abs] (2)

S4 ((smoke AND inhal*) )[Title] OR ((smoke AND inhal*) )[abs] (5 irrelevant, not downloaded)

OR/S1-S4 (54)

************************************************************************

**KSR Evidence - Kleijnen Systematic Reviews**

https://ksrevidence.com/

Searched: 8-June-2023

https://ksrevidence.com/

#1 TITLE(burn OR burns OR burned) (395)

#2 JOUNAL (burn OR burns) (218)

#2 TITLE/ABSTRACT (“burn patients” or “burns patients” or “burn care” or “burns care” or “burn related” or “burns related”) (199)

#3 TITLE/ABSTRACT ((burn OR burns OR burned) AND (wound* OR injury OR injuries OR trauma* OR “critically ill” OR “critical illness” OR scar OR scars OR infect* OR necro* OR sepsis OR septic)) 528

#4 TITLE/ABSTRACT ((burn OR burns OR burned) AND (healing OR regenerat* OR graft* OR transplant* OR surgery OR surgical OR therap* OR treatment* OR training OR rehab* OR dressing* OR supplement* OR pharma* OR nonpharma* OR intervention* OR effectiveness)) 544

#5 TITLE/ABSTRACT (postburn* OR post-burn* OR scald* OR (thermal AND injur*) OR (thermal AND wound*)) 133

#6 TITLE/ABSTRACT (smoke AND inhal* AND (burn OR burns OR burned OR injur* OR trauma* OR wound*)) 16

#7 (#1 OR #2 OR #3 OR #4 OR #5 OR #6) (726) (754)

************************************************************************

**Health Evidence**

<https://www.healthevidence.org/search.aspx>

Searched: 27 Apr 2023

Records retrieved were irrelevant (off topic or injury prevention)

#1 (burn OR burns OR burned OR scald*) (43)

#2 postburn* (0)

#3 thermal injur* (2)

#4 thermal wound*(1)

#5 smoke inhal* (10)

Zero records downloaded

********************

**DoPHER**

<https://eppi.ioe.ac.uk/webdatabases4/Search.aspx>

Searched: 27 Apr 2023

Records retrieved were irrelevant (off topic or injury prevention)

#1 (burn OR burns OR burned OR scald*) (nn)

#2 postburn* (0)

#3 thermal injur* (n)

#4 thermal wound*(n)

#5 smoke inhal* (n)

Zero records downloaded

********************
