# Supplementary material for: Exploring evidence gaps in clinical trials in thermal burns care: an umbrella review
Source: BMJ Open. 2025 Jun 25;15(6):e094303. doi: 10.1136/bmjopen-2024-094303 (PMC12198850; doi:10.1136/bmjopen-2024-094303)
Supplement: online supplemental table 1 [file bmjopen-15-6-s005.docx]

Supplementary Table 1. Characteristics of systematic reviews of interventions for burns [Grouped: Intervention type | Sorted: Reverse chronology]

| **Study ID**  **[PMID]**  **Country**  ***Title*** | **Objective(s)** | ***“Population of interest”***  **Burn size \|**  **Burn thickness \|**  **Burn cause**  **# Studies**  **# Participants** | **Interventions & comparators included**  [Network or Pairwise approach] | **Outcome(s)** | **Conclusion(s)**  **GRADE Summary of Findings: Present/Absent [# outcomes]** |
| --- | --- | --- | --- | --- | --- |
| **Reducing Pain** | | | | | |
| 1 Gillum 2022  [34534314]  Not reported  *Nonpharmacologic Management of Procedural Pain in Pediatric Burn Patients: A Systematic Review of Randomized Controlled Trials.* | "To summarize nonpharmacologic techniques used to reduce procedural pain in pediatric burn patients and quantify the efficacy of nonpharmacologic procedural pain management as an adjunct  to pediatric procedural burn care" | *“Pediatric burn patients”*  Not reported \|  Not reported \|  Not reported  Studies = 15  Participants = 424 | - Virtual reality (VR); - Directed play; - Child life therapy; - Computer tablet; - Distraction technology; - Hypnosis; - Music therapy; - Cartoons   [Pairwise] | - Pain | "This study demonstrates that nonpharmacologic therapy can be an effective adjunct in pediatric procedural burn pain management, however further studies are needed to develop standardized algorithms to integrate nonpharmacologic treatments with pharmacologic therapies."  GRADE SoF: Absent [NA] |
| 2 Luo 2019  [30480854]  USA; Netherlands; South Africa; Australia; Taiwan.  *Adjunctive virtual reality for procedural pain management of burn patients during dressing change or physical therapy: A systematic review and meta-analysis of randomized controlled trials.* | "We conducted this systematic review and meta-analysis of randomized controlled trials to verify the pain-reducing efficacy of virtual reality among burn patients undergoing dressing change or physical therapy." | *“Burn patients…during dressing change or physical therapy”*  Not reported \|  Not reported \|  Not reported  Studies = 13  Participants = 294 | - VR as an adjunct to analgesics for procedural pain control during dressing change or physical therapy sessions - No VR   [Pairwise] | - Pain - Mental health - Physical fitness/mobility - Complications (other) | "Virtual reality is an effective pain reduction measurement added to analgesics for burn patients undergoing dressing change or physical therapy. However, multicenter, parallel group design randomized controlled trials are still required."  GRADE SoF: Absent [NA] |
| 3 Norouzkhani 2022  [36426174]  USA; Canada; Netherlands; UK; Egypt; South Africa; Australia; Iran; China; South Korea.  *Effect of Virtual Reality-Based Interventions on Pain During Wound Care in Burn Patients; a Systematic Review and Meta-Analysis.* | "This systematic review and meta-analysis aimed to examine the effect of virtual reality (VR)-based interventions on pain during wound care in burn patients." | *“Burn patients during burn wound care”*  Not reported \|  Not reported \|  Not reported  Studies = 30  Participants = 1293 | - VR methods on pain of burn patients during burn wound care - No VR   [Pairwise] | - Pain | "It is suggested that health policymakers and managers equip burn wards with immersive VR devices to provide the basis for this intervention when caring for patients with burn wounds."  GRADE SoF: Absent [NA] |
| 4 Farzan 2023  [36651329]  Iran; Turkey.  *Effects of aromatherapy with Rosa damascene and lavender on pain and anxiety of burn patients: A systematic review and meta-analysis.* | "This systematic review and meta analysis aim to summarise the effects of aromatherapy with Rosa damascene (RD) and lavender on the pain and anxiety of burn patients." | *“Burn patients”*  Not reported \|  Not reported \|  Not reported  Studies = 3  Participants = 318 | - Lavender aromatherapy and inhalation - No lavender aromatherapy and inhalation   [Pairwise] | - Pain - Mental health | “Overall, this study showed that aromatherapy with RD decreased pain and anxiety of dressing procedures in burn patients. Although aromatherapy with lavender decreased pain in the patients, it was not statistically significant. More RCTs studies are required to be able to  better judge the effects of aromatherapy with RD and lavender on the pain and  anxiety of burn patients"  GRADE SoF: Absent [NA] |
| 5 de Jesus Catala 2022  [34757408]  Australia; USA.  *Virtual Reality Therapy to Control Burn Pain: Systematic Review of Randomized Controlled Trials.* | "The objective of this systematic review was to evaluate the effects of VR therapy on pain control in people who suffered burns published in the scientific literature." | *“Patients…who suffered burns regardless of the stage of the burn”*  Not reported \|  Not reported \|  Not reported  Studies = 4  Participants = 193 | - Virtual reality therapy - Cognitive therapy - Distraction techniques - Usual care   [Pairwise] | - Pain | “Virtual reality therapy has been shown to be effective in controlling pain, reducing the time spent thinking about it and greater distraction during the procedures. However, most randomized clinical trials results were not statistically significant in at least one of the moments when pain was assessed."  GRADE SoF: Absent [NA] |
| 6 Fardin 2020  [32147030]  USA; Belgium; South Africa; Iran; Taiwan; Australia.  *Non-pharmacological interventions for anxiety in burn patients: A systematic review and meta-analysis of randomized controlled trials.* | "Therefore, the present study aimed to conduct a comprehensive review of the effectiveness of NPIs on reducing anxiety in burn patients" | *“Adult burn patients”*  Not reported \|  Not reported \|  Not reported  Studies = 21  Participants = 1284 | - Music therapy - Massage - Inhalation aromatherapy - Transcranial direct current stimulation - Hypnosis - Relaxation therapy - Virtual reality - Usual care - Placebo - Stress reduction   [Pairwise] | - Pain - Mental health - Complications (other) | “Due to weak evidence, we are unable to make strong recommendations in favor of NPIs for burn anxiety. Further well-designed large sample size randomized clinical trials are warranted."  GRADE SoF: Absent [NA] |
| 7 Hadoush 2021  [35180138]  Not reported  *Non-Pharmacological Management of Burn-related Pain and Distress in Children: A Systematic Review and Meta-Analysis Study* | "This study aimed to evaluate the effect of non-pharmacological interventions including distraction, VR, hypnosis, and massage therapy on controlling pain and distress outcomes in children aged up to 18 years, and who undergoing or experienced painful procedures during burning management." | *“Children…undergoing painful burn management procedures”*  Not reported \|  Not reported \|  Not reported  Studies = 15  Participants = No reported | - Distraction techniques - Virtual reality - Hypnosis - Massage therapy - Computer games - Music therapy - Usual care - Social support   [Pairwise] | - Pain - Observer reported pain and distress | “Distraction and VR are effective non-pharmacological interventions in reducing the pain perception and distress in children during painful burn management procedures."  GRADE SoF: Present [8] |
| 8 Addab 2022  [35068011]  Not reported  *Use of virtual reality in managing paediatric procedural pain and anxiety: An integrative literature review.* | "The aims of this integrative literature review were to systematically review, appraise and synthesize the findings of studies investigating the use of VR distraction for children undergoing varying painful or anxiety-inducing medical procedures in different healthcare settings and to identify implications for research and clinical practice." | *“Children undergoing varying painful or*  *anxiety-inducing medical procedures in different healthcare settings”*  Not reported \|  Not reported \|  Not reported  Studies = 9  Participants = 224 | - Virtual reality - Virtual reality with analgesia - Analgesia - Distraction   [Pairwise] | - Pain - Mental health | “Virtual reality has the potential to help reduce the high frequency of undertreated procedural pain and anxiety among paediatric patients, ultimately reducing the fear associated with medical interventions, preventing healthcare avoidance in adulthood."  GRADE SoF: Absent [NA] |
| 9 Gates 2020  [31969473]  USA; Switzerland; Iran; Australia; Taiwan.  *Digital Technology Distraction for Acute Pain in Children: A Meta-analysis.* | "To determine the effect of digital technology distraction on pain and distress in children experiencing acutely painful conditions or procedures." | *“Children with acutely painful conditions and/or who are*  *undergoing a painful medical procedure”*  Not reported \|  Not reported \|  Not reported  Studies = 12  Participants = 528 | - Any digital technology used as a form of distraction - Usual care   [Pairwise] | - Pain - Mental health - Observer reported pain and distress | “It was impossible to determine with any certainty whether any clinically important differences in self-reported, observer-reported, or  behavioral pain might exist between the interventions."  GRADE SoF: Present [15] |
| 10 Lambert 2020  [33089901]  Iran; Australia; I USA.  *Virtual reality distraction for acute pain in children.* | "To assess the effectiveness and adverse effects of virtual reality (VR) distraction interventions for children (0 to 18 years) with acute pain in any healthcare setting." | *“Children…with acute pain in any healthcare setting”*  Not reported \|  Not reported \|  Not reported  Studies = 5  Participants = 161 | - Virtual reality interventions - Usual care   [Pairwise] | - Pain - Mental health - Physical function - Cardiovascular function - Nausea - Complications (other) | “We cannot tell whether virtual reality can reduce self-reported pain aMer a medical procedure, as we have too little confidence in the evidence available."  GRADE SoF: Present [21] |
| 11 Won 2021  [35056310]  Iran.  *Aromatherapy for Symptom Relief in Patients with Burn: A Systematic Review and Meta-Analysis.* | "This review aimed to provide an updated review of evidence  regarding the effects of aromatherapy in relieving symptoms of burn injuries, focusing on pain and  physiological distress." | *“Patients with burn injuries”*  Not reported \|  Partial and full thickness \|  Not reported  Studies = 8  Participants = 972 | - Aromatherapy inhalation - Aromatherapy massage - Placebo - Usual care   [Pairwise] | - Pain - Mental health - Sleep | “Regardless of limited evidence, our findings showed significant improvements in the management of pain and anxiety upon the usage of aromatherapy in burn patients."  GRADE SoF: Absent [NA] |
| 12 Scheffler 2018  [29287729]  USA; Iran; South Africa; Australia  *Efficacy of non-pharmacological interventions for procedural pain relief in adults undergoing burn wound care: A systematic review and meta-analysis of randomized controlled trials.* | "The aim of the present meta-analysis was to investigate the efficacy of non-pharmacological interventions for procedural pain relief in adults undergoing burn wound care compared to standard care alone or an attention control." | *“Adult patients…undergoing*  *burn wound care”*  Not reported \|  Not reported \|  Not reported  Studies = 21  Participants = 660 | - Distraction techniques - Hypnosis - Relaxation - Cognitive intervention - Usual care - Attention control   [Pairwise] | - Pain - Mental health - Use of medicines/dressings | “In summary, benefits of non-pharmacological interventions on procedural pain relief and reduction of mental distress were demonstrated. Results have been proven to be free of publication bias. However,further high quality trials are needed to strengthen the promising evidence."  GRADE SoF: Absent [NA] |
| 13 Chuan 2021  [32720308]  Not reported  *Virtual reality for acute and chronic pain management in adult patients: a narrative review.* | "This systematic narrative review evaluates clinical studies that used virtual reality in adult patients for management of acute and chronic pain." | *“Adults with acute or chronic pain”*  Not reported \|  Not reported \|  Not reported  Studies = 3  Participants = 161 | - Virtual reality - Usual care   [Pairwise] | - Pain | “These limitations suggest the evidence-base in adult patients is currently immature and more rigorous studies are required to validate the use of virtual reality as a non-pharmacological adjunct in multimodal pain management."  GRADE SoF: Absent [NA] |
| 14 Miri 2023  [36680488]  Iran  *Effects of massage therapy on pain and anxiety intensity in patients with burns: A systematic review and meta-analysis..* | "This systematic review and meta-analysis aimed to examine the effects of massage therapy on pain and anxiety intensity in patients with burns." | *“Patients with burns”*  Not reported \|  Not reported \|  Not reported  Studies = 5  Participants = 568 | - Massage - Massage with aromatherapy - Reflexology - Usual care   [Pairwise] | - Pain - Mental health | “It is recommended that health managers and policymakers pay special attention to massage therapy as a simple, low-cost, and efficient non-pharmacological treatment to relieve pain and anxiety in burn patients."  GRADE SoF: Absent [NA] |
| 15 O’Reilly 2022  [35759493]  Not reported  *Psychotherapeutic interventions for burns patients and the potential use with Stevens-Johnson syndrome and toxic epidermal necrolysis patients: A systematic integrative review.* | "The aim of this systematic integrative review was to synthesize the evidence relating to psychotherapeutic interventions used with adult burns patients and patients with SJS/TEN." | *“Adults with burns”*  Not reported \|  Not reported \|  Not reported  Studies = 9  Participants = 697 | - SMART intervention - Hypnosis - Massage - Relaxation - Virtual reality - Coping messages - Mental empowerment - Psychotherapy - Placebo hypnosis - Usual care   [Pairwise] | - Pain - Quality of life | “Following further research, some of the interventions deployed in burns patients may be applicable to SJS/TEN patients, particularly stress reduction techniques.."  GRADE SoF: Absent [NA] |
| 16 Springborg 2021  [34329326]  Not reported  *Methodology and applicability of the human contact burn injury model: A systematic review.* | "The primary aim of the present study is to systematically and critically review the methodologies and outcomes of experimental human CBI-studies." | *“Experimental studies applying a CBI-method in humans”*  Not reported \|  Not reported \|  Not reported  Studies = 37  Participants = 641 | - Antiarrhythmic agents - Gabapentinoids; - Glucocorticoids; - Glutamate receptor antagonists; - Local anesthetics; - Melatonin; - N-methyl-D-aspartate (NMDA) receptor antagonists; - Non-steroidal anti-inflammatory drugs (NSAIDs); - Opioids; - Opioid antagonists; - Placebo   [Pairwise] | - Pain - Healing - Blood tests - Biomarkers | “In conclusion, although the contact burn injury model provides robust hyperalgesia, it has limited efficacy in testing analgesic drug response. Recommendations for future use of the model are being provided, but further research is needed to improve the sensitivity of the contact burn injury method."  GRADE SoF: Absent [NA] |
| 17 Williams 2020  [32131784]  USA; Sweden; South Africa; Australia  *Efficacy and cultural appropriateness of psychosocial interventions for paediatric burn patients and caregivers: a systematic review.* | "This study assessed the effectiveness of any psychosocial intervention in reducing pain and psychological trauma, distress, and/or anxiety among paediatric burn patients and their caregivers generally. Alongside this assessment, we systematically evaluated the appropriateness and applicability of such interventions for use among Aboriginal and Torres Strait Islander families." | *“Injured children…receiving treatment at time of study, and/or their*  *caregivers.”*  Large, medium & small\|  Not reported \|  Not reported  Studies = 14  Participants = 820 | - Social skills workshop - Ditto PP - Computer games - Hypnosis - Child Life Therapy - Virtual reality - Multi modal device - Discharge preparation - Online counseling - Music therapy - Usual care - Distraction   [Pairwise] | - Pain - Mental health - Quality of life - Trauma & PTSD | “The development and assessment of psychosocial interventions to appropriately meet the needs of Aboriginal and Torres Strait Islander paediatric burn patients is required."  GRADE SoF: Absent [NA] |
| 18 Farzan 2023  [36859758]  USA; Canada; Turkey; Egypt; Iran; China; Australia.  *Effects of non-pharmacological interventions on pain intensity of children with burns: A systematic review and meta-analysis.* | "What is the effect of non-pharmacological interventions on pain intensity of children with burn? What are the effects of VR versus non-VR interventions on pain intensity of children with burn? What are the effects of non-pharmacological interventions on pain intensity of different procedures in children with burn?" | *“Children with burns.”*  Large, medium & small\|  Not reported \|  Not reported  Studies = 19  Participants = 1005 | - Virtual reality - Visual distraction - Lavender oil inhalation - Computer games - Hypnosis - Child life therapy - Ditto PP - Multi modal device - Music therapy - Usual care - Placebo   [Pairwise] | - Pain | “In sum, the result of the present study indicated that using non-pharmacological interventions significantly reduced pain intensity in children. The reduction of pain intensity was greater in  non-VR than in VR interventions. Future studies should focus on comparing VR interventions with non-VR and single versus multi-modal distraction to clarify the effectiveness of each."  GRADE SoF: Absent [NA] |
| 19 McGuiness 2011  [21880111]  Denmark; Norway; Sweden.  *A systematic review of ketamine as an analgesic agent in adult burn injuries.* | "To assess the current literature regarding the effectiveness and side-effect profile of intravenous ketamine as a means of pain relief when compared with placebo or as an adjunct to opioid analgesia in patients exposed to burn injury." | *“[Volunteers]*  *exposed to burn injury.”*  Not reported\|  Not reported \|  Not reported  Studies = 4  Participants = 106 | - Ketamine - Placebo   [Pairwise] | - Pain - Complications (other) - Side effects | “Further well-designed randomized controlled trials conducted in burn-specific populations are warranted, thus enabling the development of a relevant evidence base to support its clinical use."  GRADE SoF: Absent [NA] |
| 20 Chinchilla 2022  [34364900]  Not reported  *Efficacy of opioids and non-opioid analgesics in the treatment of post procedure pain of burned patients: a narrative review.* | "The objective of this narrative review is to evaluate the efficacy of opioids, non-steroidal antiinflammatory drugs, paracetamol, gabapentinoids, ketamine, and lidocaine in the treatment of acute pain in burn victims." | *“burn patients with clinical*  *treatment indications and/or second-degree burns or higher.”*  Large and medium\|  Partial and full thickness\|  Not reported  Studies = 6  Participants = 397 | - Gabapentin; - Lidocaine; - Fentanyl; - Nalbuphine; - Ketamine; - Intranasal fentanyl, - Placebo - Saline; - PCA-fentanyl; - Morphine; - Dexmedetomidine   [Pairwise] | - Pain - Mental health - Physical function - Delirium - Complications (other) | “Evidence of efficacy is very limited. Fentanyl, nalbuphine, and ketamine seem to be effective for controlling acute pain in burn patients, whereas gabapentin and lidocaine did  not show any efficacy."  GRADE SoF: Absent [NA] |
| 21 Czech 2022  [35490982]  Not reported  *Virtual reality intervention as a support method during wound care and rehabilitation after burns: A systematic review and meta-analysis.* | "This systematic review and meta-analysis aimed to analyze and synthesize the evidence on the effectiveness of virtual reality (VR) interventions in the prevention of pain, fear and anxiety during burn wound care procedures." | *“Patients…undergoing burn wound care”*  Not reported\|  Not reported\|  Flame, contact & scald  Studies = 17  Participants = Not reported | - Virtual reality - Usual care - Distraction   [Pairwise] | - Pain - Mental health - Cardiovascular function - Physical fitness/mobility - Healing - Observer rated pain and distress | “VR seems to be an effective therapeutic support in burn wound care procedures for reducing pain. However, this systematic review and meta-analysis highlights the need for more research into the use of VR as a distraction method."  GRADE SoF: Absent [NA] |
| 22 Eijlers 2019  [31136330]  Not reported  *Systematic Review and Meta-analysis of Virtual Reality in Pediatrics: Effects on Pain and Anxiety.* | "In this meta-analysis, we will collate evidence on the effectiveness of VR as either a distraction or an exposure tool, compared to standard care, on pain and anxiety in pediatric patients undergoing medical procedures." | *“Pediatric patients ≤21 years of age undergoing medical procedures”*  Not reported\|  Not reported\|  Not reported  Studies = 6  Participants = 198 | - Virtual reality - Usual care   [Pairwise] | - Pain - Mental health | “Large effect sizes indicate that VR is an effective distraction intervention to reduce pain and anxiety in pediatric patients undergoing a wide variety of medical procedures. However, further research on the effect of VR exposure as a preparation tool for medical procedures is needed because of the paucity of research into this field."  GRADE SoF: Absent [NA] |
| 23 Grunzweig 2020  [33215037]  Not reported  *Regional Anesthetic Blocks for Donor Site Pain in Burn Patients: A Meta-Analysis on Efficacy, Outcomes, and Cost.* | "This study is intended to evaluate the efficacy of regional anesthesia in the burn population to decrease narcotic consumption and to assess the impact on hospitalization costs." | *“Adult patients with burn injuries who underwent split-thickness skin grafts”*  Not reported\|  Not reported\|  Not reported  Studies = 2  Participants = 101 | - Continuous regional anesthesia; - Single shot regional anesthesia - No analgesic blocks   [Pairwise] | - Pain - Physical function - Narcotic side effects - Complications (other) - Cost of treatment - Use of medicines/dressings | “Regional anesthesia at skin graft donor sites significantly decreases narcotic consumption in burn patients. Regional anesthesia is cost-effective, decreases side effects, and may result in shorter hospital stays due to improved pain management."  GRADE SoF: Absent [NA] |
| 24 Joseph 2022  [35225720]  Not reported  *Effects of Slow Deep Breathing on Acute Clinical Pain in Adults: A Systematic Review and Meta-Analysis of Randomized Controlled Trials.* | "The primary aim of this systematic review and meta-analysis is to investigate the effects of SDB on acute pain." | *“Adult patients with acute, experimental, and chronic pain.”*  Not reported\|  Not reported\|  Not reported  Studies = 2  Participants = 98 | - Deep breathing exercises - Usual care   [Pairwise] | - Pain | “Meta-analysis of post-intervention pain scores demonstrated that SDB was associated with significantly lower pain scores compared with a control group, but with high levels of heterogeneity. Subgroup analyzes demonstrated that trials of burn pain were associated with a larger reduction in pain which partially explains the heterogeneity. Very low certainty evidence suggests that SDB may reduce acute pain intensity."  GRADE SoF: Present [3] |
| 25 Kilic 2021  [33821879]  USA; Netherlands; Taiwan; Australia  *Using Virtual Technology for Fear of Medical Procedures: A Systematic Review of the Effectiveness of Virtual Reality-Based Interventions.* | "This review aims to collate evidence for the impact of VR on fear of medical procedures." | *“People who were reported to experience fear, anxiety, or pain during medical procedures.”*  Not reported\|  Not reported\|  Not reported  Studies = 7  Participants = 233 | - Virtual reality - Usual care   [Pairwise] | - Pain - Mental Health | “Overall, the effectiveness of VR used for the application of distraction among burn wound care type of medical procedures consistently demonstrates improved pain outcomes, though results are mixed in its ability to reduce anxiety."  GRADE SoF: Absent [NA] |
| 26 McBride 2022  [36133279]  Not reported  *Systematic literature review of topical local anesthesia or analgesia to donor site wounds.* | "This study is designed to address the question of which agent, if any, is favoured over the others and whether there are any safety data regarding their use." | *“People of any age in any setting who have a DSW created as a result of harvesting a STSG.”*  Not reported\|  Not reported\|  Not reported  Studies = 11  Participants = 550 | - Bupivacaine; - EMLA; - Morphine; - Lidocaine - Lidocaine - Usual care   [Pairwise] | - Pain - Blood tests - Burn wound infections - Use of medicines/dressings | “Topical local anesthetics (lidocaine or bupivacaine) provide good analgesia, both during and after STSG harvest, at well below toxic serum levels, but there are no good data  determining the best local anesthetic agent to use. There is no evidence morphine performs better than placebo."  GRADE SoF: Absent [NA] |
| 27 Ridout 2021  [34185015]  USA; Australia.  *Effectiveness of Virtual Reality Interventions for Adolescent Patients in Hospital Settings: Systematic Review.* | "The aim of this review was to systematically identify available evidence regarding the use of virtual reality interventions  for adolescent patients in hospital settings to evaluate effectiveness, suitability, and safety and identify opportunities for future  research." | *“Adolescent patients in hospital settings.”*  Not reported\|  Not reported\|  Not reported  Studies = 2  Participants = 69 | - Virtual reality - Usual care   [Pairwise] | - Pain | “Virtual reality can provide a safe and engaging way to reduce pain and anxiety in adolescents while in hospital, particularly when virtual reality software is highly immersive and specifically designed for therapeutic purposes."  GRADE SoF: Absent [NA] |
| 28 Sajeev 2021  [34185015]  USA; Australia.  *Interactive video games to reduce paediatric procedural pain and anxiety: a systematic review and meta-analysis.* | "We aimed to evaluate the effect of interactive video game interventions on children’s procedural pain and anxiety,  including the effect of different types of video games on those outcomes." | *“Children and*  *adolescents (18 yr) who had undergone a painful procedure..”*  Not reported\|  Not reported\|  Not reported  Studies = 8  Participants = 363 | - Virtual reality - Usual care   [Pairwise] | - Pain - Mental health - Satisfaction - Healing - Complications (other) - Use of medicine/dressings - Length of procedure | “Our findings support introducing easily available video games, such as distraction-based conventional video games, into routine practice to minimise paediatric procedural pain and child/caregiver anxiety."  GRADE SoF: Absent [NA] |
| **Improving wound management** | | | | | |
| 29 Chaganti 2019  [31000315]  USA; China.  *A systematic review of foam dressings for partial thickness burns.* | "The primary objective of this systematic review is to compare the rate of re-epithelialization (complete healing) and time to wound healing of two treatment options, foam dressings versus silver sulfadiazine with gauze, in patients of all ages who have sustained second degree thermal burns." | *“Patients with partial thickness burns.”*  Small and medium\|  Partial and full thickness\|  Not reported  Studies = 3  Participants = 346 | - Foam dressing - Traditional silver-sulfadiazine - Non-foam dressing   [Pairwise] | - Pain - Healing - Burn wound infections | “Moderate quality evidence indicates that there is no significant difference in wound healing between silver-containing foam dressing and SSD dressing. However, foam has the added benefit of reduced pain during the early treatment phase and potentially decreased infection rates."  GRADE SoF: Absent [NA] |
| 30 Hoogewerf 2020  [32725896]  Not reported.  *Topical treatment for facial burns.* | "To assess the effects of topical interventions on wound healing in people with facial burns of any depth." | *“People of any age with a facial burn wound of any degree in any care setting.”*  Medium\|  Partial thickness\|  Not reported  Studies = 12  Participants = 507 | - Any remedy, agent, substance, device or skin substitute (biological or bioengineered) - No intervention - Placebo   [Pairwise] | - Pain - Quality of life - Healing - Burn wound infections - Scar texture, colour and healing - Length of hospital stay - Follow-up surgery | “There is mainly low to very low-certainty evidence on the effects of any topical intervention on wound healing in people with facial burns."  GRADE SoF: Present [17] |
| 31 Huang 2021  [33127309]  Netherlands; China.  *Platelet-rich plasma for the treatment of burn wounds: A meta-analysis of randomized controlled trials.* | "The purpose of this study was to perform a comprehensive search of the current literature and to conduct a meta-analysis of RCTs to determine the efficacy and safety of PRP in the treatment of burn wounds." | *“Patients diagnosed with burn, including different burn sites and depths.”*  Not reported\|  Not reported\|  Not reported  Studies = 8  Participants = 539 | - Local injection or dressing with platelet-rich plasma - Silver-sulfadiazine - Placebo - Saline - Usual care   [Pairwise] | - Appearance - Quality of life - Healing - Complications (other) - Scar texture, colour and healing | “In conclusion, this meta-analysis indicated that topical PRP treatment on burn wounds can improve wound healing rate, shorten healing  time and reduce the incidence of adverse events. However, further research is needed to standardize the preparation and use of PRP and to evaluate the long-term clinical outcome of PRP in the treatment of burn  wounds."  GRADE SoF: Present [6] |
| 32 Kwa 2019  [31515194]  Not reported.  *A systematic review on surgical and nonsurgical debridement techniques of burn wounds.* | "To provide a complete overview of all burn debridement techniques  studied in recent literature and to find the best evidence with regard to efficiency and safety." | *“Burn etiology of thermal origin.”*  Not reported\|  Not reported\|  Not reported  Studies = 4  Participants = Not reported | - Hydrosurgery - Enzymatic debridement - Conventional tangential excision   [Network] | - Healing - Mortality - Scar texture, colour and healing - Costs of treatment - Length of hospital stay - Grafting surgery - Re-operation - Excision time - Graft take - Reconstructions | “Although CTE is considered the gold standard for burn debridement, we found limited evidence on efficiency and safety of this technique. More recently, several relatively new debridement techniques (HS and ED) have been described in studies of increasingly methodological quality. These studies show promising results with regard to relevant modern burn wound outcomes such as need for grafting and scar quality."  GRADE SoF: Absent [NA] |
| 33 Nimia 2019  [29903603]  Not reported.  *Comparative study of Silver Sulfadiazine with other materials for healing and infection prevention in burns: A systematic review and meta-analysis.* | "The aim of this systematic review with meta-analysis was to compare the effect of Silver  Sulfadiazine (SSD) with other new dressings, with or without silver, on healing and infection  prevention in burns." | *“Burn patients.”*  Medium\|  Partial and full thickness\|  Not reported  Studies = 24  Participants = 1769 | - Silver Sulfadiazine - Other topical treatments   [Pairwise] | - Healing - Burn wound infections | “Considering the clinical trials conducted up to the present time, the authors concluded that new dressings with and without silver show better results than SSD for wound healing, and burns treated with dressings without silver are less likely to become infected than burns with SSD. No differences between SSD and new silver materials were observed in relation to infection prevention."  GRADE SoF: Absent [NA] |
| 34 Salehi 2020  [32424404]  Not reported.  *Clinical Value of Debriding Enzymes as an Adjunct to Standard Early Surgical Excision in Human Burns: A Systematic Review.* | "The aim of this study was to investigate clinical trials using debriding agents for burn wound in humans in a systematic review" | *“Burn patients.”*  Small and medium\|  Partial and full thickness\|  Not reported  Studies = 9  Participants = 607 | - Bromelain - Collagenase - Papain - Sutilains - Nexobrid - Hydrocolloid dressings - Surgery   [Network] | - Quality of life - Healing - Complications (other) - Scar texture, colour and healing - Length of hospital stay - Blood transfusion - Surgery | “Surgical excision still remains the standard of care for burn wounds debridement. Further randomized clinical trials with multicentric design and larger sample size could more clearly  elucidate the role of promising agents such as Bromelain as adjunct to standard surgical treatment for burn wounds."  GRADE SoF: Absent [NA] |
| 35 Givol 2019  [31145533]  France.  *A systematic review of Calendula officinalis extract for wound healing.* | "This systematic review evaluates the role of C. officinalis flower  extract on wound healing in vivo compared to controls." | *“Included were primary research studies investigating the use of C. officinalis extract as a monotherapy in wound healing.”*  Not reported\|  Partial and full thickness\|  Not reported  Studies = 1  Participants = 156 | - Topical calendula extract ointment - Chemical debridement - Usual care   [Pairwise] | - Pain - Healing - General infections - Complications (other) | “This review identified some evidence for the beneficial effects of C. officinalis extract  for wound healing, consistent with its role in traditional medicine. There is a need for larger, well-designed randomized control trials to assess the effect of calendula on wound healing including complications."  GRADE SoF: Absent [NA] |
| 36 Saeg 2021  [34181622]  Not reported.  *Evidence-Based Nutritional Interventions in Wound Care.* | "The four specific objectives of this review are to (1) conduct a comprehensive search of the  published literature on nutritional interventions in wound healing, (2) outline the nutrients used in the  identified studies, (3) analyze outcomes, and (4) propose evidence-based guidelines." | *“Patients undergoing nutritional intervention during wound care.”*  Large\|  Not reported\|  Not reported  Studies = 2  Participants = 53 | - Vitamins C & E with zinc - Copper, selenium and zinc - Placebo   [Pairwise] | - Healing - Burn wound infections - Sepsis - Length of hospital stay - Regrafting | “Burn wounds benefit from oral vitamin A, vitamin B1, vitamin B6, vitamin B12, vitamin D, vitamin E, zinc, parenteral calcium, copper, magnesium, selenium, and zinc supplementation."  GRADE SoF: Absent [NA] |
| 37 Yang 2021  [33284236]  USA; Macedonia; Iran; Egypt; India; Bangladesh.  *Efficacy and feasibility of amniotic membrane for the treatment of burn wounds: A meta-analysis.* | "We aimed to evaluate the role of AM dressings in burn wounds." | *“Burns irrespective of the degree of burn.”*  Not reported\|  Not reported\|  Not reported  Studies = 11  Participants = 816 | - Amniotic membrane - Usual care - Honey-impregnated gauze - Antimicrobial ointments - Silver sulfadiazine ointment - Silver sulfadiazine gauze - Polyurethane membrane - Skin staples   [Pairwise] | - Healing - Burn wound infections - Scar texture and healing - Costs of treatment - Use of medicines/dressings | “Amniotic membrane has beneficial effects in treating burn wounds; however, the evidence needs to be strengthened by further robust randomized controlled trials."  GRADE SoF: Absent [NA] |
| 38 Csenkey 2019  [31553768]  Not reported.  *Systemic antibiotic prophylaxis does not affect infectious complications in pediatric burn injury: A meta-analysis.* | "The present meta-analysis aimed at determining whether systemic antibiotic prophylaxis prevents infectious complications in pediatric patients with burn injuries." | *“Children with burn injury.”*  Not reported\|  Not reported\|  Not reported  Studies = 1  Participants = 80 | - Prophylactic antibiotics - Unclear   [Pairwise] | - Burn wound infections - General infections | “Our findings provide quantitative evidence for the inefficacy of systemic antibiotic prophylaxis in preventing infections in pediatric burns. To validate our conclusion, multinational, randomized trials in a diverse population of children with burn injuries are warranted."  GRADE SoF: Absent [NA] |
| 39 Jalkh 2022  [36127967]  USA.  *Oxandrolone Efficacy in Wound Healing in Burned and Decubitus Ulcer Patients: A Systematic Review.* | "This systematic review aims to analyze previously conducted randomized controlled trials to evaluate the evidence of the applicability of oxandrolone therapy." | *“Adult patients with burns and adult patients with pressure ulcers.”*  Small medium and large\|  Not reported\|  Not reported  Studies = 5  Participants = 208 | - Oxandrolone - Placebo   [Pairwise] | - Healing | “Analysis of the available data demonstrated a significant advantage in skin healing using oxandrolone in adult burn patients as an adjunct."  GRADE SoF: Absent [NA] |
| 40 Ziegler 2020  [33215038]  Israel; Italy; China; Australia.  *Evidence and Trends in Burn Wound Debridement: An Evidence Map.* | "To summarize the evidence and trends for eschar removal by burn wound debridement currently available, an evidence map as variant of the systematic review, was prepared." | *“Severely burned patients.”*  Small medium and large\|  Partial thickness\|  Not reported  Studies = 5  Participants = 202 | - Enzymatic debridement - Collagenase gel - Versajet debridement - Lubricant and razor debridement - Excisional debridement - Vaseline - Escharectomy - Debridement with sterile gauze   [Pairwise] | - Pain - Healing - General infections - Complications (other) - Scar texture, colour and healing - Further surgical interventions | “Enzymatic debridement has been shown to be an effective tool for early eschar removal and in addition reduces the need for autografting of the debrided burn wound with a relatively high level of evidence (LoE 2-). Wound debridement by means of hydrosurgery is more precise compared to conventional wound excision and preserves viable dermis, but a positive effect on wound healing or scar formation could not been shown (LoE 2). Furthermore, rarely reported techniques comprise larvae therapy, debridement by laser, and other technical adjuncts, but the level of evidence is limited (LoE 4-/5)."  GRADE SoF: Absent [NA] |
| 41 Toleubayev 2021  [33948167]  Germany.  *Efficacy of erythropoietin for wound healing: A systematic review of the literature.* | "To systematically review the available literature on the efficacy of erythropoietin for wound healing in human patients." | *“Human patients [wound healing].”*  Not reported\|  Not reported\|  Not reported  Studies = 1  Participants = 84 | - Subcutaneous erythropoietin - Placebo   [Pairwise] | - Healing | “Results presented by Gunter and colleagues were rather inconclusive due to a high withdrawal rate (64 out of 84 patients missed the primary endpoint)."  GRADE SoF: Absent [NA] |
| 42 Jiang 2021  [34564840]  Not reported.  *Silicone gel sheeting for treating hypertrophic scars.* | "To assess the effects of silicone gel sheeting for the treatment of hypertrophic scars in any care setting." | *“People with any hypertrophic scars.”*  Not reported\|  Not reported\|  Not reported  Studies = 9  Participants = Unclear | - Silicone gel - Silicone elastomer - Silicone gel sheets - Silicone gel bandage - Placebo - Plastic   [Pairwise] | - Pain - Healing | “The relative effects of SGS compared with alternative treatments in many of the comparisons is unclear: it is often uncertain whether the SGS assessed in these (all single, small) trials is associated with any difference in scar severity. The certainty of this evidence is low or very low, primarily due to study limitations and levels of imprecision around the estimates of effect."  GRADE SoF: Present [49] |
| **Improving burns resuscitation and early management** | | | | | |
| 43 Glas 2020  [31202528]  Not reported.  *Changes in ventilator settings and ventilation induced lung injury in burn patients-A systematic review.* | "The aim of this systematic review was to investigate and describe changes in ventilation practice in burn patients over time. We also aimed to investigate the  occurrence of VILI in ventilated burn patients." | *“Adult or pediatric burn and/or inhalation injury patients receiving mechanical ventilation during ICU stay.”*  Not reported\|  Not reported\|  Not reported  Studies = 3  Participants = 161 | - High frequency percussive ventilation - Conventional mechanical ventilation - Volumetric diffusive respiration - Low tidal volume ventilation - High frequency percussive ventilation - Pressure control ventilation   [Pairwise] | - Time on mechanical ventilator - Mortality | “This systematic review, which provides an overview of the existing literature on MV in burn patients, shows a high variety in MV practices in this patient population, reflecting the lack of  consensus regarding the optimal ventilation strategy. A trend towards implementation of lung protective MV is noticeable, however it remains unclear whether burn patients benefit from  these strategies."  GRADE SoF: Absent [NA] |
| 44 Griffin 2022  [35688782]  South Korea.  *The effect of 20 minutes of cool running water first aid within three hours of thermal burn injury on patient outcomes: A systematic review and meta-analysis.* | "What effect does the application of CRW for 20 min within three  hours of burn injury have on patient outcomes, and is it more effective than alternative remedies, or no CRW?" | *“Children…and adults… with thermal burns (flame, scald, contact) with any thickness and total body surface area percentage involvement.”*  Not reported\|  Partial thickness\|  Scald  Studies = 1  Participants = 33 | - Tap water for 20 minutes - Burn cool spray - Burnshield   [Pairwise] | - Pain - Healing - Skin temperature | “There is considerable evidence to recommend 20 min of CRW within three hours of injury as the gold standard of first aid for thermal burns. International consensus is required."  GRADE SoF: Absent [NA] |
| 45 Hassoun-Kheir 2021  [32686565]  USA; Iran.  *The Effect of beta-Blockers for Burn Patients on Clinical Outcomes: Systematic Review and Meta-Analysis.* | "*We aimed to assess the effects of b-blockers administration in hospitalized patients with burns*." | *“Children and adults with burn injury who were treated in a burn*  *center, including hospitalized patients.”*  Medium and large\|  Not reported\|  Not reported  Studies = 4  Participants = Not reported | - Any b-blocker, administered at any dose, duration of treatment, or route of administration. - Placebo - No treatment   [Pairwise] | - Healing - Mental health - Quality of life - Blood tests - Body weight/weight loss - Bone strength - Cardiovascular function - Blood loss - General infections - Sepsis - Mortality - Complications (other) - Length of hospital stay | “In summary, the available evidence on clinical outcomes of b-blocker use after burn injury is limited. No reduction in mortality, length of stay, or infections was observed with propranolol in our review. The current data do not support nor refutes an advantage to b-blockers after burns."  GRADE SoF: Present [6] |
| 46 Valentini 2019  [31618360]  USA; South Africa; Iran.  *Pediatric enteral nutrition therapy for burn victims: when should it be initiated?* | "To review the scientific evidence regarding the initiation of enteral nutrition in the pediatric burn population." | *“Pediatric patients with burns.”*  Medium and large\|  Not reported\|  Not reported  Studies = 3  Participants = 781 | - EEN (< 24 hours) - EEN (< 24 hours) plus enteral volume replacement - EEN (3 - 6 hours) plus enteral volume replacement - LEN (> 48 hours) - LEN (> 48 hours) plus conventional IV volume replacement - LEN (> 48 hours) plus conventional IV volume   replacement  [Pairwise] | - Blood tests - Body weight/weight loss - Mortality - Complications (other) - Length of hospital stay | “Analysis of the different intragroup variables suggests the importance of starting nutritional support early. Considering the number of pediatric burn patients, there is a need  for robust studies with greater scientific  impact."  GRADE SoF: Absent [NA] |
| 47 McQuilten 2018  [28803752]  USA.  *Optimal Dose, Timing and Ratio of Blood Products in Massive Transfusion: Results from a Systematic Review.* | "The aim of this systematic review was to assess the effect of dose, timing and ratio of blood component therapy (FFP, platelets, cryoprecipitate or fibrinogen concentrate) to RBCs on morbidity, mortality and transfusion in critically bleeding patients requiring massive  transfusion." | *“A patient population (pediatric and/or adult) who had*  *critical bleeding and had received, or was anticipated to receive, a massive transfusion and measured at least one outcome of interest (mortality, morbidity, transfusion requirements or quality of life).”*  Medium and large\|  Not reported\|  Not reported  Studies = 1  Participants = 45 | - 1:1 (FFP:RBC) - 1:4 (FFP:RBC)   [Pairwise] | - Transfusion volumes | “On the limited evidence available, there is insufficient basis to recommend a 1:1:1 over a 1:1:2 ratio or standard care for adult patients with critical bleeding requiring massive transfusion."  GRADE SoF: Present [7] |
| 48 Pu 2018  [30222632]  Not reported.  *Early Enteral Nutrition Reduces Mortality and Improves Other Key Outcomes in Patients With Major Burn Injury: A Meta-Analysis of Randomized Controlled Trials.* | "To identify, appraise, and synthesize current evidence  to determine whether early enteral nutrition alters patient outcomes from major burn injury." | *“Patients with major burn injuries.”*  Small, medium and large\|  Not reported\|  Not reported  Studies = 7  Participants = 527 | - Early enteral nutrition - Delayed nutritional intake   [Pairwise] | - Organ function - Sepsis - Mortality - Complications (other) - Length of hospital stay - Gastrointestinal hemorrhage - Pneumonia - Renal failure | “The improvements in clinical outcomes demonstrated in this meta-analysis are consistent with the physiologic rationale cited to support clinical recommendations for early  enteral nutrition made by major clinical practice guidelines: gut integrity is preserved leading to fewer gastrointestinal hemorrhages, less infectious complications, a reduction in consequent organ failures, and a reduction in the onset of sepsis."  GRADE SoF: Absent [NA] |
| 49 Lewis 2018  [30073665]  Canada; USA; Switzerland; Denmark; UK.  *Colloids versus crystalloids for fluid resuscitation in critically ill people.* | "To assess the effect of using colloids versus crystalloids in critically ill people requiring fluid volume replacement on mortality, need for blood transfusion or renal replacement therapy (RRT), and adverse events (specifically: allergic reactions, itching, rashes).." | *“Critically ill people who required fluid volume replacement in hospital or emergency out-of-hospital settings.”*  Not reported\|  Partial and full thickness\|  Not reported  Studies = 8  Participants = 455 | - Colloids - Crystalloids   [Pairwise] | - Blood tests - Body weight/weight loss - Organ function - General infections - Sepsis - Mortality - Graft success | “Using colloids (starches; dextrans; or albumin or FFP) compared to crystalloids for fluid replacement probably makes little or no difference to the number of critically ill people who die. It may make little or no difference to the number of people who die if gelatins or crystalloids are used for fluid replacement."  GRADE SoF: Present [32] |
| 50 Fuentes 2019  [31684690]  USA.  *Early enteral nutrition (within 48 hours) versus delayed enteral nutrition (after 48 hours) with or without supplemental parenteral nutrition in critically ill adults.* | "To evaluate the efficacy and safety of early enteral nutrition (initiated within 48 hours of initial injury or ICU admission) versus delayed enteral nutrition (initiated later than 48 hours a3er initial injury or ICU admission), with or without supplemental parenteral nutrition, in critically ill adults." | *“Critically ill adults.”*  Not reported\|  Not reported\|  Not reported  Studies = 1  Participants = 27 | - Early enteral nutrition with supplemental parenteral nutrition - Delayed nutrition with supplemental parenteral nutrition   [Pairwise] | - Burn wound infections - Mortality - Length of hospital stay - Duration of mechanical ventilation | “Overall, results showed no clear differences in the number of deaths within 30 days (one study, 38 participants), intolerance to feeding (one  study, 59 participants), or development of pneumonia (four studies, 192 participants), between those who received early enteral nutrition or delayed enteral nutrition. We assessed the evidence as very low-quality, meaning the findings could potentially change with additional studies."  GRADE SoF: Present [6] |
| 51 Cook 2020  [31775021]  USA; Brazil; Iran; Germany.  *Opportunities for Palliative Care in Patients With Burn Injury-A Systematic Review.* | "We performed a systematic review of existing evidence concerning the palliative care needs of burn patients." | *“Adults sustaining burn injury.”*  Medium and large\|  Not reported\|  Not reported  Studies = 5  Participants = 641 | - Pre-discharge training program - Outpatient care coordinator - Individualized self-care program - Group CBT - Usual care   [Pairwise] | - Mental health - Quality of life | “Most existing palliative care-related research in burn patients addresses interventions for physical symptoms with minimal literature concerning other domains. Opportunities exist for further research of palliative care in burn  populations with emphasis on addressing interventions for all domains and better standardizing the language and outcomes  for the palliative care interventions."  GRADE SoF: Absent [NA] |
| 52 Kao 2018  [29024269]  USA; Canada; Switzerland; Albania; Italy; UK; China.  *Fluid Resuscitation in Patients With Severe Burns: A Meta-analysis of Randomized Controlled Trials.* | "We conducted a systematic review and meta-analysis to compare the efficacies of hyperosmotic and isoosmotic solutions in restoring hemodynamic stability after burn injuries." | *“Patients with burn injuries.”*  Medium and large\|  Not reported\|  Not reported  Studies = 10  Participants = 502 | - Hyperosmotic - HES - Isoosmotic - Plasma - Albumin   [Pairwise] | - Blood tests - Urine output - Mortality - Complications (other) - Length of hospital stay - ARDS | “Hyperosmotic fluid resuscitation appears to be an attractive choice for severe burns in terms of TBSA or burn depth. Further investigation is recommended before conclusive recommendation."  GRADE SoF: Absent [NA] |
| 53 Heliste 2022  [35838226]  USA; Pakistan.  *Beta-blocker treatment in the critically ill: a systematic review and meta-analysis.* | "To assess the effect of treatment with b-blockers in critically ill adults, we conducted a systematic review and meta-analysis of randomized controlled trials." | *“Critically ill adults.”*  Medium and large\|  Not reported\|  Not reported  Studies = 3  Participants = 26 | - Beta-blockers (Propranolol) - Placebo - Usual care   [Pairwise] | - Quality of life - Organ function - Mortality | “In this systematic review we found that b-blocker treatment reduced mortality in critical illness. Use of b-blockers in critical illness thus appears safe after initial hemodynamic stabilization."  GRADE SoF: Present [8] |
| 54 Mortada 2023  [36578449]  Canada; UK; India; China.  *The Effects of Glutamine Supplementation on Reducing Mortality and Morbidity among Burn Patients: A Systematic Review and Meta-analysis of Randomized Controlled Trials.* | "We aim to study the role of GLN supplements in decreasing mortality, length of hospitalization (LOH), and infection in severely burned patients." | *“Burn patients who received either parenteral or enteral GLN supplement.”*  Medium and large\|  Not reported\|  Flame, contact and scald  Studies = 7  Participants = 328 | - Glutamine supplementation - No glutamine supplementation   [Pairwise] | - Blood tests - Organ function - General infections - Mortality - Length of hospital stay | “GLN supplementation has been linked to lower hospital mortality and infection-related morbidity in burn patients. Furthermore, larger-scale and higher-quality studies are needed to assess whether there are any statistically and clinically significant changes."  GRADE SoF: Absent [NA] |
| 55 Haynes 2021  [XXXXXXX]  Not reported.  *Hyper-oncotic vs. Hypo-oncotic Albumin Solutions: a Systematic Review of Clinical Efficacy and Safety* | "This systematic review aimed to evaluate evidence on the efficacy and safety of hyper-oncotic vs. hypo-oncotic albumin solutions across different clinical settings." | *“Patients receiving volume therapy.”*  Not reported\|  Not reported\|  Not reported  Studies = 1  Participants = Not reported | - 2.5% albumin infusion - Unclear   [Pairwise] | - Lung water | “In contrast, an RCT by Goodwin et al. showed that 2.5% albumin infusion leads to accumulation of lung water, suggesting that crystalloids alone may lead to better outcomes in this patient population."  GRADE SoF: Present [8] |
|  |  |  |  |  |  |
| **Improving rehabilitation** | | | | | |
| 56 Edger-Lacoursiere 2023  [35662480]  Not reported.  *Rehabilitation interventions after hand burn injury in adults: A systematic review.* | "This review aims to summarise and evaluate what rehabilitation interventions are most effective in improving hand function, range of motion (ROM), hand strength, scar  outcome, return to work, level of impairment/disability, level of burn knowledge and decreasing edema following hand burns in adult burn survivors." | *“Adults aged 18 years or older who sustained hand burns.”*  Not reported\|  Not reported\|  Not reported  Studies = 14  Participants = 1238 | - Dynamic orthosis - Continuous passive motion - Compression - Burn educational and training - Resistance training - Motor imagery - Virtual reality - Dynamic Orthosis - Electric stimulation - Silicone pressure garment - Ultrasound therapy - Paraffin therapy - No orthosis - Usual care - Static Orthosis - Pressure garment only - Passive exercise   [Pairwise] | - Pain - Quality of life - Appearance - Physical function - Physical fitness/mobility - Scar texture, colour and healing - Edema | “This review supports the clinical practice of the following interventions: 1) The use of adhesive compression wraps for patients who have increased edema to increase hand function and ROM; 2) The use of compression (adhesive compressive wrap, compression bandage or intermittent compression pump) to decrease hand edema following burn injury; 3) Participating in general rehabilitation to increase hand function and patient perceived level of disability; 4) The use of an orthosis to increase ROM and a dynamic MCP orthosis to increase hand function; 5) If available, incorporate the use of VR based rehabilitation to increase hand function and hand strength; 6) The use of paraffin to increase hand PROM; 7) The use of gels to reduce hand scar thickness; 8) The use of an education component in rehabilitation to increase the level of burn knowledge."  GRADE SoF: Absent [NA] |
|  |  |  |  |  |  |
| 57 Hampton 2021  [33722450]  USA.  *Evaluation of high protein intake to improve clinical outcome and nutritional status for patients with burns: a systematic review.* | "To evaluate the evidence for improved nutritional status and clinical outcomes of burn patients following the administration of a high protein intake." | *“Adult or paediatric…patients with burns (in acute or rehabilitative stages) with any percentage TBSA.”*  Medium and large\|  Not reported\|  Not reported  Studies = 2  Participants = Unclear | - High protein: 25% of kcal from protein (4.9g/kg/d). Normal hospital diet and oral diet supplemented with whey protein - Group 1: 1.31.5g/kg/d protein. Group 2: 2g/kg/d protein. - Usual care   [Pairwise] | - Blood tests - Body weight/weight loss - Physical fitness/mobility - Burn wound infections - Length of hospital stay | “There is currently only very weak evidence to justify administering high protein diets to patients following burns."  GRADE SoF: Absent [NA] |
|  |  |  |  |  |  |
| 58 Yoshida 2022  [36544605]  USA.  *A systematic review assessing the effectiveness of hand therapy programmes in adults with burns using the International Classification of Functioning, Disability and Health framework.* | "This systematic review summarized the effectiveness of different hand therapy programmes in adults with burns using the ICF framework of outcomes in the three phases. We hypothesized that there is evidence on the effectiveness of rehabilitation programmes in all phases, in terms of the quality of life (QOL) (health conditions), body functions and structures, activity, participation, and environmental factors, in the ICF framework." | *“Patients aged*  *18 years or older, with burns on the upper extremity, hand and finger.”*  Small, medium and large\|  Not reported\|  Not reported  Studies = 10  Participants = Unclear | - Conventional rehabilitation - Physical rehabilitation - Motor imagery - Xbox Kinect - Massage therapy - Extracorporeal shock wave therapy - Orthosis therapy - Unclear   [Pairwise] | - Pain - Mental health - Quality of life - Physical function - Healing - Physical fitness/mobility - Scar texture, colour and healing | “This systematic review demonstrated that hand therapy programmes including conventional rehabilitation substantially contributed to improve burn-related issues in adult patients with burn."  GRADE SoF: Absent [NA] |
|  |  |  |  |  |  |
| 59 Flores 2018  [29320878]  USA; Egypt; Australia.  *Exercise training for improving outcomes post-burns: a systematic review and meta-analysis.* | "To determine the effectiveness of different modalities of exercise and to evaluate the safety  of exercise-based interventions post-burns." | *“Adults and children at any stage of burn injury and of any burn percentage.”*  Not reported\|  Not reported\|  Not reported  Studies = 17  Participants = Unclear | - Pure aerobic - Aerobic plus resistance - Whole body vibration - Isokinetic - Coordination and strength - Range of motion (RoM) - Video game–assisted exercises - Unclear   [Pairwise] | - Pain - Quality of life - Blood tests - Body weight/weight loss - Cardiovascular function - Complications (other) - Surgical release of contractures | “Limited evidence suggests that exercise has a beneficial effect on physical and physiological  outcomes in patients post-burn. Further trials using high-quality methodology are required, with focus on reporting of adverse events, health-related quality of life and psychological outcomes."  GRADE SoF: Absent [NA] |
|  |  |  |  |  |  |
| 60 Gittings 2020  [32340772]  USA; Egypt.  *Corrigendum to 'Resistance training for rehabilitation after burn injury: A systematic literature review & meta-analysis' [Burns 44 (2018) 731-751].* | "The objective was to determine the effectiveness of resistance training on muscle strength, lean mass, function, quality of life and pain, in children and adults after burn injury." | *“Adults and children at any stage of burn injury and of any burn percentage.”*  Not reported\|  Not reported\|  Not reported  Studies = 9  Participants = 295 | - Resistance training - No resistance training   [Network] | - Physical function - Body weight/weight loss - Physical fitness/mobility | “Further research with robust methodology is recommended to assess the potential benefit suggested in this review."  GRADE SoF: Present [20] |
|  |  |  |  |  |  |
| 61 Retouvey 2018  [29931288]  USA.  *The Impact of Time of Mobilization After Split Thickness Skin Graft on Lower Extremity Wound Healing-Systematic Review and Meta-analysis.* | "The aim of this systematic review and metaanalysis is to summarize the evidence for timing of mobilization in the extremities after STSG." | *“The study population was defined as adults…with wounds in the UE or LE from any etiology treated with*  *STSG.”*  Not reported\|  Not reported\|  Not reported  Studies = 1  Participants = Unclear | - Early ambulation - Unclear   [Pairwise] | - Healing - Graft loss | “We conclude that early mobilization may be the preferred strategy for patients treated with STSG to the LE as it provides good graft healing and prevents morbidity. High-quality studies are needed to provide more robust recommendations on mobilization in the LE. Studies are needed to provide evidence in the UE."  GRADE SoF: Absent [NA] |
|  |  |  |  |  |  |
| 62 Sha 2021  [34331107]  USA; Egypt.  *The effects of resistance training on children with burns: a meta-analysis.* | "The aim of this meta-analysis was to provide information on evidence-based medicine  for clinical selection of RT for rehabilitation program of  children with burns." | *“Burned children, no cause, extent or location*  *of burns restriction was applied.”*  Large\|  Full thickness\|  Not reported  Studies = 12  Participants = 379 | - Resistance training - Usual care   [Pairwise] | - Physical function - Body weight/weight loss - Cardiovascular function - Muscle strength and endurance | “In this review, moderate- to low-quality evidence showed that RT has a positive effect on muscular strength, lean body mass, cardiopulmonary function, gait parameters and psychological quality of life in children with burns. However, the current quality of evidence for these results in literature on burn rehabilitation is poor."  GRADE SoF: Absent [NA] |
|  |  |  |  |  |  |
| 63 Shneider 2020  [31504622]  Not reproted.  *Systematic Review and Expert Consensus on the Use of Orthoses (Splints and Casts) with Adults and Children after Burn Injury to Determine Practice Guidelines.* | "The objective of this review was to systematically evaluate the available literature addressing the use of orthoses (splints and casts) with adult and pediatric burn survivors and determine whether practice guidelines could be  proposed." | *“Adults*  *who have sustained a burn injury.”*  Small, medium and large\|  Not reported\|  Not reported  Studies = 5  Participants = 164 | - Dynamic orthosis - Multi- axis orthosis - No orthosis - Reduced time using orthosis   [Pairwise] | - Quality of life - Physical function - Complications (other) | “Due to the low level of evidence in the available literature, only one practice guideline could be recommended: orthotic use should be considered as a treatment choice for improving range of motion or reducing contracture in adults who have sustained a burn injury."  GRADE SoF: Absent [NA] |
|  |  |  |  |  |  |
|  |  |  |  |  |  |
| **Improving scarring** | | | | | |
| 64 Buhalog 2021  [32926192]  Not reported.  *Ablative fractional laser treatment of hypertrophic burn and traumatic scars: a systematic review of the literature.* | "Evaluating the efficacy of ablative fractional lasers for the management of HTS, provide appropriate context, identify critical gaps, and inform a path forward." | *“Subjects with hypertrophic scars incurred from burns and related trauma were considered.”*  Not reported\|  Not reported\|  Not reported  Studies = 4  Participants = 76 | - Lutronic eCO2 3 Tx, 4–6 WA - Lumenis UltraPulse and Lumenis M22 Intense Pulsed Light. 4 Tx, 6–8 WA - Candela CO2RE 3 Tx - Unclear   [Pairwise] | - Pain - Itch - Appearance - Healing - Scar texture and healing | “There is abundant existing literature on the use of AFLs in the management of HTS but study heterogeneity limits generalizability."  GRADE SoF: Absent [NA] |
|  |  |  |  |  |  |
| 65 De Decker 2022  [35367089]  Not reported.  *The use of fluid silicone gels in the prevention and treatment of hypertrophic scars: a systematic review and meta-analysis.* | "This review aims to provide the most comprehensive overview of the available literature on fluid silicone gels, as well as a meta-analysis on its efficacy in improving scar quality and incidence." | *“[Patients undergoing treatment of] fluid silicone gels on scars from all wound aetiologies, combined with a quality assessment and meta-analysis.”*  Not reported\|  Not reported\|  Not reported  Studies = 7  Participants = 425 | - Fluid silicone gels - Placebo gels - Silicone gel sheets - Pressure therapy - Methylprednisolone - Aloe vera gel   [Pairwise] | - Quality of life - Appearance - Vancouver scar scale - Scar texture, colour and healing - Chromometry - Profilometry - DermoatoSpectrometer | “Our meta-analysis clearly showed significant scar improvement in terms of pliability, pigmentation, height and scar quality overall. However, improvements in vascularity were not unequivocal. The efficacy of silicone gel alone compared to the efficacy of other standard skin care products is largely unexplored and should be the subject of future high quality randomized trials."  GRADE SoF: Absent [NA] |
|  |  |  |  |  |  |
| 66 Killey 2021  [33305622]  USA; Canada; Taiwan; China.  *Effectiveness of interventions for optimising adherence to treatments for the prevention and management of scars: A systematic review.* | "This systematic review aimed to examine the effectiveness of adherence interventions utilised in adults and children with, or at risk of developing scarring and determine the types of adherence  interventions used and the theoretical frameworks  these interventions were based on." | *“The population included adults and children using treatments to prevent or manage scarring following skin wounds (e.g. burn injury, surgery, lacerations, piercings, vaccinations,*  *acne, other conditions affecting the skin).”*  Not reported\|  Not reported\|  Not reported  Studies = 4  Participants = 57 | - Pressure garments - Silicone gel sheeting - Exercises - Scar management education   [Pairwise] | - Adherence to treatment - Physical fitness/mobility - Healing - Scar texture, colour and healing | “Adherence interventions using education or technology for people receiving burn scar treatment may improve adherence. Further studies are needed particularly in children, with a focus on including outcomes of importance to patients (e.g. quality of life) and identifying core components of effective adherence interventions using theoretical frameworks."  GRADE SoF: Absent [NA] |
|  |  |  |  |  |  |
| 67 Badwy 2022  [34637585]  Not reported.  *Electrical neurostimulation for the treatment of chronic pruritus: A systematic review.* | "The primary aim of this study was to investigate the evidence of efficacy of electrical neurostimulation in the treatment of chronic itch conditions." | *“Patients suffering from refractory chronic itch.”*  Not reported\|  Not reported\|  Not reported  Studies = 1  Participants = 30 | - Transcutaneous electrical nerve stimulation (TENS) - Usual care   [Pairwise] | - Itch | “Our review indicates that electrical neurostimulation could be considered for the treatment of refractory chronic itch of selected aetiologies, such as atopic dermatitis or burn pruritus. However, better understanding of the mechanisms of action of the neurostimulation modalities and regimens in various pruritic conditions is necessary."  GRADE SoF: Absent [NA] |
|  |  |  |  |  |  |
| 68 Wigley 2023  [36226364]  Germany; Belgium; Egypt; Saudi Arabia; South Korea.  *Shock Wave Therapy in Plastic Surgery: A Review of the Current Indications.* | "Herein, we explore the evidence for ESWT to provide tangible therapies in plastic surgery and outline the key mechanisms in which these occur." | *“Patients suffering from refractory chronic itch.”*  Not reported\|  Not reported\|  Not reported  Studies = 8  Participants = 383 | - Extracorporeal Shock Wave Therapy - Placebo - Usual care   [Pairwise] | - Pain - Quality of life - Healing - Itch - Appearance - Physical function - Blood tests - Scar texture, colour and healing - Surgical debridement - Grafting | “There is yet insufficient evidence to support the effectiveness of any specific intervention included in this review; however, all included studies reported improvements in key outcomes."  GRADE SoF: Absent [NA] |
|  |  |  |  |  |  |
| 69 Yanhui 2022  [35691021]  Belgium; Spain; Egypt; South Korea; China.  *Safety and efficacy of treating post-burn pathological scars with extracorporeal shock wave therapy: A meta-analysis of randomised controlled trials.* | "The objective of this systematic review and meta-analysis is to quantitatively evaluate the efficacy and safety of extracorporeal shockwave therapy  combined with comprehensive rehabilitation therapy on post-burn pathological scars  compared to comprehensive rehabilitation therapy alone." | *“Patients with post-burn pathological*  *scars.”*  Not reported\|  Not reported\|  Not reported  Studies = 9  Participants = 422 | - Extracorporeal Shock Wave Therapy - Placebo - Usual care   [Pairwise] | - Pain - Itch - Healing - Physical function - Complications (other) - Scar texture, colour and healing | “Available data preliminarily suggested that the combination of extracorporeal shockwave therapy and comprehensive rehabilitation therapy had better therapeutic effect on post burn pathological scars than comprehensive rehabilitation therapy alone, without obvious side effects. However, further clinical well-controlled randomised controlled trials are needed."  GRADE SoF: Absent [NA] |
|  |  |  |  |  |  |
| 70 Nischwitz 2020  [32506727]  Not reported.  *Evidence-based therapy in hypertrophic scars: An update of a systematic review.* | "This review has been conducted to investigate the current state  of available high-quality studies for the treatment of hypertrophic  scars." | *“Hypertrophic scars in living humans.”*  Not reported\|  Not reported\|  Not reported  Studies = 9  Participants = 660 | - Triamcinolone - Silicone gel - Lotion with massage - Pressure therapy - Co2 laser - Triamcinolone with Fluorouracil - Verapamil - Placebo - Lotion - Silicone gel - Pressure therapy - No co2 laser   [Pairwise] | - Pain - Physical function - Scar texture, colour and healing | “This systematic review showed that still few high-quality studies exist to evaluate therapeutic means and their mechanisms for hypertrophic scars. Among these, most of them assessed the efficacy of intralesional triamcinolone injections with the same treatment protocol. Intralesional injection appears to be the best option for  hypertrophic scar treatment."  GRADE SoF: Absent [NA] |
|  |  |  |  |  |  |
| **Improving surgical interventions** | | | | | |
| 71 Battistini 2023  [36516423]  Not reported.  *Topical Hemostatic Agents in Burn Surgery: A Systematic Review.* | "This systematic review aims to report on all topical hemostatic agents described in burn surgery, their efficacy at controlling intra-operative bleeding, as well as complications associated with their use." | *“Acute burn surgery patients.”*  Not reported\|  Not reported\|  Not reported  Studies = 4  Participants = Unclear | - Phenylephrine (1:20,000) - Epinephrine (1:200,000); - Tranexamic Acid (4 mg/ml) - Thrombin (100 IU/ml); - Epinephrine (1:50,000)   [Pairwise] | - Intra-operative blood loss | “A multitude of topical hemostatic agents has been reported in the burn literature, with a wide range of dosages and modes of deliveries, as well as protocolization with other blood conservation techniques to limit blood loss during surgery. Determining the optimal topical hemostatic agent is limited by low-quality data and challenges with consistent reporting of intra-operative blood loss and other clinically meaningful outcomes."  GRADE SoF: Absent [NA] |
|  |  |  |  |  |  |
| 72 Clare 2022  [36047788]  Not reported.  *Amniotic membrane transplantation for acute ocular burns.* | "To compare the effect of AMT with medical therapy in the first seven days after an ocular surface burn, compared to medical therapy alone." | *“Study participants of any age who had an acute chemical or thermal ocular burn of severity grade II or worse.”*  Small\|  Not reported\|  Not reported  Studies = 2  Participants = 128 | - One or more AM patches to cover the cornea and the whole or part of the ocular surface - Usual care   [Pairwise] | - Physical function - Healing - Complications (other) | “There is uncertain evidence to support the treatment of moderate acute ocular surface burns with AMT in addition to standard medical  therapy as a means of preventing failure of epithelialisation by day 21, improving visual outcome and reducing corneal neovascularisation, symblepharon formation and time-to-epithelialisation. For severe burns, the available evidence does not indicate any significant benefit of treatment with AMT."  GRADE SoF: Present [12] |
|  |  |  |  |  |  |
| 73 Wardhana 2022  [37016595]  Not reported.  *Efficacy Of Skin Substitutes For Management Of Acute Burn Cases: A Systematic Review.* | "This systematic review is designed to weigh the efficacy of skin substitutes compared to standard treatment for managing  acute burn cases." | *“All types of burn, any location of burn wound, and burn experienced by children as well as adults.”*  Small, medium and large\|  Partial and full thickness\|  Not reported  Studies = 13  Participants = 431 | - Biobrane - TransCyte - Integra - Glyaderm - Suprathel and Apligraft - Silver Sulfadiazine - Biobrane - Duoderm - Bacitracin - Allograft   [Network] | - Pain - Healing - Scar texture, colour and healing - Length of hospital stay - Number of dressing changes | “All of the skin substitutes studied exhibited at least non-inferior to superior performance compared to standard treatment in terms of efficacy in treating acute burn wounds, not limited to burn depth, size, location or patient age."  GRADE SoF: Absent [NA] |
|  |  |  |  |  |  |
| 74 Bairagi 2021  [33941398]  USA; Italy; Switzerland; Australia; China.  *A systematic review and meta-analysis of randomized trials evaluating the efficacy of autologous skin cell suspensions for re-epithelialization of acute partial thickness burn injuries and split-thickness skin graft donor sites.* | "The primary objective of this review was to determine the effectiveness of ASCS when compared to non-ASCS usual treatment on time to wound re-epithelialization of acute partial thickness burn injuries and split-thickness skin graft donor site wounds." | *“Humans of all age groups, partial thickness burn injuries and*  *split-thickness skin graft donor site wounds.”*  Not reported\|  Not reported\|  Not reported  Studies = 5  Participants = 347 | - Autologous skin cell suspension - Autologous skin cell suspension plus Platelet - Autologous skin cell suspension plus Biobrane - Split-thickness skin graft - Platelet rich plasma - Paraffin gauze - Hydrocolloid dressing - Silver or biological dressing   [Pairwise] | - Pain - Itch - Appearance - Physical function - Healing - Burn wound infections - Complications (other) - Scar colour - Costs of treatment - Surgery within 10 days | “Compared to standard care, ACSC may reduce pediatric partial thickness burn wound and adult split-thickness skin graft donor site TTRE."  GRADE SoF: Present [10] |
|  |  |  |  |  |  |
| 75 Miller 2019  [30724361]  Not reported.  *Systematic review of fibrin glue in burn wound reconstruction.* | "This systematic review evaluated outcomes of fibrin glue compared with conventional SSG attachment techniques." | *“Patients with burn wounds.”*  Not reported\|  Not reported\|  Not reported  Studies = 2  Participants = 178 | - Fibrin glue - Staples   [Pairwise] | - Pain - Patient satisfaction - Complications (other) - Costs of treatment | “As the evidence is sparse, the quality very low and the risk of bias significant both within and across studies, it is not possible to make any recommendations regarding the use of fibrin glue in burn wounds."  GRADE SoF: Present [9] |
|  |  |  |  |  |  |
| 76 Miroshnychenko 2021  [XXXXXXXXX]  USA; Denmark; Iran; Egypt; India.  *Comparison of early surgical intervention to delayed surgical intervention for treatment of thermal burns in adults: A systematic review and meta-analysis* | "To determine if early surgical intervention (excision and grafting within 7 days) improves outcomes when compared to delayed surgical intervention (excision and grafting after 7 days) in adults  with thermal burns." | *“Adult patients with thermal*  *burns of all degrees and body surface areas.”*  Small, medium and large\|  Not reported\|  Not reported  Studies = 9  Participants = 512 | - Early excision - Early grafting - Delayed excision - Delayed grafting   [Pairwise] | - Appearance - Physical function - Healing - Blood transfusions - Mortality - Scare texture, colour and healing - Length of hospital stay - Repeat surgeries | “Early excision and grafting may reduce mortality and improve other patient important outcomes in adults with thermal burns, however most outcomes are based on low or very low certainty evidence."  GRADE SoF: Present [11] |
|  |  |  |  |  |  |
| **Improving psychosocial outcomes** | | | | | |
| 77 Hornsby 2020  [31697370]  USA; Switzerland; Sweden; Iran; Australia.  *Psychosocial Interventions Targeting Recovery in Child and Adolescent Burns: A Systematic Review.* | "The objective of this review was to identify and critically evaluate the types of psychosocial interventions, outcome measures utilized, and quality of comparative studies directed at supporting the psychosocial recovery of pediatric burn survivors." | *“Children…or parents of children who suffered a burn injury.”*  Not reported\|  Not reported\|  Not reported  Studies = 16  Participants = 987 | - Cognitive based therapy - Art therapy - Social work contact - Counseling - Psychologist or psychiatrist support - Support groups - School adjustment - Unclear   [Pairwise] | - Pain - Mental health - Sleep | “The majority focus on distraction techniques in the acute recovery phase delivered in the form of VR. These interventions were found to be effective in reducing pain and anxiety prior to or during burn dressing changes or during physical therapy for a wide range of pediatric ages. Burn camps, cognitive behavioral therapy, and parent  counseling are promising but more large-scale, robust studies are needed to determine the effectiveness of interventions."  GRADE SoF: Absent [NA] |
|  |  |  |  |  |  |
| 78 Hutchinson 2022  [35349676]  USA; Iran; Saudi Arabia.  *Sleep Quality Among Burn Survivors and the Importance of Intervention: A Systematic Review and Meta-Analysis.* | "1) characterize measured sleep quality among burn survivors, using a systematic literature review approach and 2) investigate how interventions may be used to influence sleep quality in patients with burn injuries, using a meta-analytic approach." | *“Individuals with burn injuries (inpatients and outpatients).”*  Not reported\|  Partial and full thickness\|  Not reported  Studies = 16  Participants = 987 | - Melissa Tea - Haloperidol or zolpidem - Exercise - Joint mobilisation - Relaxation - Reflexology - Betamethasone - Avena Sativa Extract - Eucerin Ointment - Diphenhydramine - Black tea - Haloperidol or zolpidem - Usual care   [Pairwise] | - Sleep - Polysomnography | “This review demonstrates that sleep is negatively affected in burn survivors. Furthermore, the use of interventions shows a large positive effect on sleep quality thus, emphasizing their potential in clinical  practice. However, there exists a paucity of interventions aimed at improving sleep in patients with burn injuries. Considering the importance of optimized sleep, a greater number of interventions, both behavioral and pharmacological, must be evaluated in this population."  GRADE SoF: Absent [NA] |
|  |  |  |  |  |  |
| 79 Paggiaro 2022  [35361563]  USA; Brazil; Australia.  *Posttraumatic stress disorder in burn patient: A systematic review.* | "The aim of this study was to conduct a systematic review by assessing PTSD therapeutic measures in burn patients and their effectiveness in reducing symptoms or prevention in patients  with different age ranges." | *“Burn patients.”*  Small and medium\|  Not reported\|  Not reported  Studies = 8  Participants = 688 | - Coping with Accident Reactions - Sertraline - Medical hypnosis - Safety, Meaning, Activation and Resilience Training - Education program - Propranol - Placebo - Usual care - Nondirective Supportive Pyschotherapy   [Network] | - Pain - Mental health - Quality of life | “Medications (sertraline and propanolol) were not effective in reducing stress symptoms. Four studies used cognitive-behavioral therapies, which achieved the best results for PTSD improvement in burn patients. Hypnosis and an informational education program were also evaluated and did not show success in reducing PTSD."  GRADE SoF: Absent [NA] |
|  |  |  |  |  |  |
| 80 Kudchadkar 2022  [35703367]  USA.  *Non-pharmacological interventions for sleep promotion in hospitalized children.* | "To evaluate the effect of non-pharmacological sleep promotion interventions in hospitalized children and adolescents on sleep quality  and sleep duration, child or parent satisfaction, cost-effectiveness, delirium incidence, length of mechanical ventilation, length of stay, and mortality." | *“Infants, children, and adolescents…admitted to the hospital for more than 48 hours.”*  Not reported\|  Not reported\|  Not reported  Studies = 1  Participants = 10 | - Healing touch - Usual care   [Pairwise] | - Sleep - Sleep via polysomnography | “The included studies were heterogeneous, so we could not quantitatively synthesize the results.Our narrative summary found inconsistent, low to very low-certainty evidence. Therefore, we are unable to determine how non-pharmacologic sleep promotion interventions affect sleep quality or sleep duration compared with usual care or other interventions."  GRADE SoF: Present [14] |
|  |  |  |  |  |  |
| **Prevention** | | | | | |
| 81 Garcia-Diaz 2023  [36833860]  Australia.  *Utility of Telehealth Platforms Applied to Burns Management: A Systematic Review.* | "This study aims to critically evaluate the literature on the cost–benefit impact of TH in burn patients and to investigate the clinical effectiveness of implementing TH strategies." | *“Patients requiring treatment of their burns with a high degree of*  *specialisation, particularly in tertiary hospitals.”*  Not reported\|  Not reported\|  Not reported  Studies = 1  Participants = 498 | - Telehealth interventions - No telehealth interventions   [Pairwise] | - Knowledge of burns prevention - Costs of treatment | “Given the low cost and large reach of smartphone apps to deliver content to and engage with targeted populations, smartphone apps can be used for widespread injury prevention campaigns and public health campaigns generally. However, it is important  to acknowledge that a change in knowledge does not necessarily reflect a change in behaviour."  GRADE SoF: Absent [NA] |
|  |  |  |  |  |  |
| 82 Tupetz 2020  [33373371]  South Africa.  *Prevention of childhood unintentional injuries in low- and middle-income countries: A systematic review.* | "This project aims to delineate the childhood injury prevention initiatives in LMICs." | *“Children in LMICs.”*  Not reported\|  Not reported\|  Not reported  Studies = 2  Participants = Unclear | - Education and provision of safety devices - Unclear   [Pairwise] | - Knowledge of safety practices | “Increased attention and funding are required to go beyond educational initiatives with self reported measures and little follow-up time to robust interventions that will reduce the global  burden of unintentional injuries among children."  GRADE SoF: Absent [NA] |
|  |  |  |  |  |  |
| 83 Price 2021  [34729373]  South Africa; Malawi; Rwanda; Ethiopia; Iran; China.  *Burn injury prevention in low- and middle- income countries: scoping systematic review.* | "The objective of this scoping review and narrative synthesis was to summarise and understand the initiatives that have been carried out to reduce burn injuries in LMIC and their effectiveness." | *“Participants of all genders and ages were*  *included in this study.”*  Not reported\|  Not reported\|  Not reported  Studies = 8  Participants = 21,505 | - Education programme - Training program - Replacement of open fire stoves with closed stoves - No intervention   [Pairwise] | - Knowledge of burn prevention - Injury risk score - incidents of burns | “There is a lack of published literature describing large-scale burn prevention programmes in LMIC that can demonstrate sustained reductions in burn incidence. Population level, collaborative projects are necessary to drive forward burn prevention through specific environmental or legislative changes and supplementary educational programmes."  GRADE SoF: Absent [NA] |
|  |  |  |  |  |  |
|  |  |  |  |  |  |
|  |  |  |  |  |  |
|  |  |  |  |  |  |
|  |  |  |  |  |  |
|  |  |  |  |  |  |
|  |  |  |  |  |  |
