# Supplementary material for: Exploring evidence gaps in clinical trials in thermal burns care: an umbrella review
Source: BMJ Open. 2025 Jun 25;15(6):e094303. doi: 10.1136/bmjopen-2024-094303 (PMC12198850; doi:10.1136/bmjopen-2024-094303)
Supplement: online supplemental appendix 1 [file bmjopen-15-6-s006.docx]

**Reliability Assessment**

**CRITERION 1: Did the authors describe the eligibility criteria for studies to be included or excluded from the review?**

[Yes; No; Unsure]

**CRITERION 2: Overall, in your judgment and with all the presented information (e.g. search terms, number of trials identified), do you think that the search for evidence was reasonably comprehensive?**

[Yes; No]

Consider aspects such as how many databases were searched, the timing of the search, efforts to search non-bibliographic/grey literature sources, how many search results were returned given the scope of the question. In general, a "comprehensive" search should involve at minimum (1) an electronic search of two or more bibliographic databases; (2) using a search strategy comprising a mixture of controlled vocabulary and keywords; (3) report using at least one other method of searching such as searching of conference abstracts; identified ongoing trials; complemented electronic searching by hand search methods (e.g., checking reference lists); and contacted included study authors or experts. If "No" is selected, please provide your rationale

**CRITERION 3: Did the author(s) report assessing the risk of bias or methodological quality using any method in individual studies?**

[Yes; No; Unsure; Not applicable]

Check "Yes," "No," "Can't tell," or "Not applicable." Many methods exist to assess the risk of bias, including scales, in which various components of quality are scored and combined to give a summary score; or checklists, in which specific questions are asked; or domain-based evaluation, in which critical assessment are made separately for different domain, for example, allocation concealment, making etc. Select "Not applicable" if the review included zero studies.

**a. Did the author(s) conduct any quantitative analyses (i.e., meta-analyses/meta-regression)?**

[Yes; No; Not applicable]

Select "Not applicable" if the review included zero studies. If there are no meta-analyses in the review (i.e., 'No') or the review included zero studies (i.e., 'Not applicable'), then CRITERION 4 is not applicable and is skipped (automatically built into the form).

**CRITERION 4: Was the quantitative analysis appropriate?**

[Yes, the meta-analyses appeared appropriate; No, the meta-analyses were inappropriate]

Consider all aspects of the analyses in whether this was appropriate or not. CLINICAL, STATISTICAL, & DESIGN HETEROGENEITY: Were the studies and outcomes similar enough within a comparison to justify combining the studies using meta-analyses? STATISTICAL MODEL & APPROACH: Did the authors use an appropriate statistical model (e.g., random effects vs. fixed effects) and did they meta-analyze treatment effects across studies without breaking randomization (i.e., meta-analyzing by treatment arms)? If you believe the analyses are inappropriate, please specify why

**CRITERION 5: In your judgment, do you think the conclusions related to the main research question of the systematic review are supported by the data?**

[Yes; No; Not applicable]

Consider the overall results that have been presented in the systematic review and the discussion/conclusions that have been made. Are the conclusions appropriate for the findings? If very strong conclusions are made about the safety or effectiveness of interventions and recommendations for practice, are these justified? If not, please specify why you do not believe they are appropriate

**Are there any other reasons why you might consider the review to be unreliable?**

[Yes; No reason]

Consider whether there are any aspects of the review that might influence the overall product. For example, if the review is sponsored by the intervention's producer or the authors are employees of the parent company that makes the intervention, does the overall review seem fair in considering the five elements considered above, or is there a bias? Or, are there potential errors or inconsistencies in some aspects of the review? If there are any other reasons why you do not trust this review, please specify them

**Overall, in your judgement, do you think the systematic review is reliable?**

[Yes; No]

A review MUST MEET ALL FIVE criteria (and not have any "Other" reasons for being unreliable) to be considered reliable. If ANY of Questions 4 - 9 indicate that there is a reason for being unreliable, this review is considered to be not reliable. IF THE ANSWER TO THIS QUESTION IS "NO" (i.e., the review is not reliable), END HERE AND DO NOT CONTINUE WITH PICO EXTRACTION

**Data extraction items (Complete for all papers that pass criteria assessment)**

**Q1. What is the objective of the systematic review?**

[Free text box]

Record VERBATIM the objective of the systematic review described in one of the following sections of the article using this order of priority: Abstract, Methods, Background, Other. If not reported, insert NR in the text box

**Q2. What is the main conclusion of the systematic review?**

[Free text box]

Record VERBATIM the main conclusion(s) of the systematic review described in one of the following sections of the article using this order of priority: Abstract, Methods, Background, Other. If not reported, insert NR in the text box

**Q3. In your judgment, did the author(s) ask at least one clearly-focused research question?**

[Yes; No; Unsure]

Check “Yes” if at least the population and test intervention are specified in the review

**Q4. Basic review characteristics**

4a. How many RCTs were included?

[Free text; Unclear; Not stated]

4b. How many participants were included across studies?

Only extract if the number is obviously reported, otherwise select Unclear or Not stated

[Multiple choice (single response) for ranges 1-50; 55-100 etc; Unclear; Not stated]

4c. What countries (and regions) were the studies conducted in?

Only extract if the number is obviously reported, otherwise select Unclear or Not stated

[Multiple choice (multiple responses) for global regions North America; South America; Europe; Africa; Asia; Oceania; Low-resource setting; Unclear; Not stated]

**Q5.** **What is the population of interest for the systematic review?**

[Free text]

Please copy and paste the VERBATIM description of the population that is of interest for this review

**Q6. What is the intervention and comparator of interest for the systematic review?**

Please copy and paste the VERBATIM description of all interventions and comparators that are of interest for this review. If the review uses a network approach (i.e., compares all interventions with each other, select this option in Q13a3

Q6a. Intervention

Q6b. Comparator

Q6c Network approach [Yes (i.e. all interventions and controls of interest are compared with each other); No (i.e. only pairwise comparisons are made between intervention(s) and controls)]

**Q7. Please specify the intervention(s) and comparator(s) according to the following categories.**

Please specify the types of interventions and controls that are compared in this systematic review

[Multiple choice (multiple responses) of interventions Resuscitation and fluid management; Burns size and depth calculations; Pain management; Pharmalogical pain management; Non-pharmalogical pain management; Wound management; Dressings and wound covers; Skin grafts; Skin donor sites; Skin substitutes; Surgery; Reconstructive/Plastic surgery; Infection control; Infection management; Scar management; Scar-reducing interventions; Scar itch intervention; Inhalation injury; Psychosocial interventions; Rehabilitation; Physiotherapy; Compression therapy; Exercise; Nutrition; Prevention; Other please state]

[Multiple choice (multiple responses) of possible comparators, including Usual/standard care; Placebo; No treatment; Delayed treatment/wait list; Active control etc]

**Q8. What outcomes are included in this review?**

[Multiple choice (multiple responses) of potential outcomes]

A systematic review may specify many different outcomes of interest in the methods, some of which may not have corresponding results. Please use the checkboxes to specify which outcomes had results that were reported in the review. For example, a review may plan to assess visual function, but if no results are presented for visual function, it should not be selected from this list

**Q9. Does the paper include GRADE assessment or Summary of Results table?**

[Yes; No]

Q9a [If Yes] How many comparison/outcome pairs/rows are reported?

[Free text]

**Q10. Please specify the population according to the following categories.**

[Checklist of burns populations,]

[Adults; children; Large burn; Medium burn; Small burn; Partial thickness burn; Full thickness burn; Flame burn; Contact burn; Scald]

**Q11. Please specify which intervention categories the intervention(s) fall within.**

For each type of intervention that was studied in this systematic review, specify the sub-type and the specific intervention. (Categories were generated from analysis of survey responses indicating which areas of burns treatment are most important to patients, carers and clinicians.)

1. Improving psychosocial outcomes

2. Improving scarring

3. Managing long term issues and chronic morbidities related to burns injuries

4. Improving wound management

5. Improving rehabilitation

6. Reducing pain

7. Optimising access to treatment

8. Improving burns resuscitation and early management

9. Improving surgical interventions for burns

10. Clinician & patient/carer interactions and communications

11. Improving clinician and patient education around burns treatment

12. Inhalation injury

13. Optimising the timings of treatments

14. Prevention

15. Resources

16. Clinician’s wellbeing

17. Other (Developing new treatments; Standardising care)

**Q12. Do you have any comments or notes about this review?**

[Free text]

Is there anything that should be noted about this review such as it being in a non-English language, only being an abstract, not having a full-text, or being potentially ineligible?
