# Supplementary material for: Exploring evidence gaps in clinical trials in thermal burns care: an umbrella review
Source: BMJ Open. 2025 Jun 25;15(6):e094303. doi: 10.1136/bmjopen-2024-094303 (PMC12198850; doi:10.1136/bmjopen-2024-094303)
Supplement: online supplemental appendix 2 [file bmjopen-15-6-s007.docx]

| Theme/Category | Details |
| --- | --- |
| 1. Improving Psychosocial Outcomes | Psychological impact of the injury, trauma/PTSD, scarring, long-term impact. The social impact of scars; impact on family and social factors e.g. work and education; psychological interventions; social, cultural attitudes and stigma. |
| 2. Improving scarring | Management of scars including compression garments, minimisation of scaring, laser and silicone treatments, skin sensitivity, hypertrophic scars, contractures, itching and temperature regulation. |
| 3. Managing long-term and chronic issues related to burns | Long-term issues including chronic conditions, impact on mobility and activities, or specific body areas. Growing and/or aging with scars. |
| 4. Improving wound management | Wound dressings, management of wounds (prior to scar formation). Infection diagnosis, treatment and prevention. Debridement and cleaning of burn wounds. |
| 5. Improving burns rehabilitation | Rehabilitation including physiotherapy and occupational therapy, nutrition, acute and long-term rehabilitation. |
| 6. Reducing pain | Pain management, the impact of pain, analgesia and chronic pain. |
| 7. Optimising access to burns treatment | Access to specialist burns services. Equity of access to burns care. |
| 8. Improving burns resuscitation and early management | Resuscitation protocols and formulae for fluid resuscitation. Calculations for burns surface area. First aid and pre-hospital treatment. Medication (not related to analgesia). Patient mortality. |
| 9. Improving surgical intervention in burns care | All aspects of surgery and surgical interventions, including excision and reconstruction. Skin grafts and skin substitutes. |
| 10. Health care professional and patient/carer interactions and communications | Clinical interactions and communication between HCPs and patients/carers. |
| 11. Improving health care professionals and patient's education about burns care | Clinician and patient education regarding burns care, including public and community education. |
| 12. Inhalation injury | Inhalation injury diagnosis and treatment |
| 13. Optimising the timing of burn care | Timings and sequence of surgery for wound management and skin grafts. Timings of mobilisation for rehabilitation. |
| 14. Burns prevention | Prevention of burn injuries, including within specific populations |
| 15. Burns care resources | Clinical resources and facilities for burns treatments. |
| 16. Clinician's wellbeing | The impact of burns care on clinician’s wellbeing, e.g. vicarious trauma. |
| 17 Developing new treatments and standardising care | New treatments and technologies. Standardisation of burns care. |

Broad research priorities identified from Priorities in Global Burns Research Priorities Setting Partnership from survey data exploring stakeholders’ experiences of burn care.
