# Supplementary material for: Exploring evidence gaps in clinical trials in thermal burns care: an umbrella review
Source: BMJ Open. 2025 Jun 25;15(6):e094303. doi: 10.1136/bmjopen-2024-094303 (PMC12198850; doi:10.1136/bmjopen-2024-094303)
Supplement: Supplementary file 8 [file bmjopen-2024-094303.reviewer_comments.pdf]

PEER REVIEW HISTORY

BMJ Open publishes all reviews undertaken for accepted manuscripts. Reviewers are asked to complete a checklist review form and are provided with free text boxes to elaborate on their assessment. These free text comments are reproduced below.

ARTICLE DETAILS

Title (Provisional)

Exploring evidence gaps in clinical trials in thermal burns care: An umbrella review.

Authors

Richards, Hollie Sarah; Staruch, Robert; Lee, Alice; Meirte, Jill; Stiles, Krissie; Martin, Niall; Savović , Jelena; Qureshi, Riaz; Kinsella, Suzannah; Dawson, Sarah; Evans, Janine; Dheansa, Baljit; Blazeby, Jane

---

VERSION 1 - REVIEW

**Reviewer**	**1**
**Name**	**Dvorak, Justin**
**Affiliation**	**MetroHealth Medical Center**
**Date**	**24-Oct-2024**
**COI**	**None**

The authors take on the important task of identifying the strength of the Burn literature that we turn to today to help us take care of burn patients. Anyone who practices burn surgery in some form or another knows that the evidence for some of our management is not necessarily as strong as it is for other surgical specialties. The authors performed a well-done umbrella review of the current literature and identified the areas of reliable evidence as well as areas where reliable evidence is lacking. One minor revision needed is on page 2, line 37. The sentence just stops without completing the statement.

**Reviewer**	**2**
**Name**	**Buta, Martin**
**Affiliation**	**Massachusetts General Hospital, Division of Plastic and Reconstructive Surgery**
**Date**	**18-Dec-2024**
**COI**	**None**

Very comprehensive, well-executed, and much needed umbrella review that will be an excellent resource for researchers and practitioners in burn care interested in developing well-designed, high impact studies.
